# A cell-free platform for the prenylation of natural products and application to cannabinoid production

**DOI:** 10.1038/s41467-019-08448-y

**Published:** 2019-02-04

**Authors:** Meaghan A. Valliere, Tyler P. Korman, Nicholas B. Woodall, Gregory A. Khitrov, Robert E. Taylor, David Baker, James U. Bowie

**Affiliations:** 1 Department of Chemistry and Biochemistry, Molecular Biology Institute, UCLA-DOE Institute, University of California, Los Angeles, 90095 CA USA; 2 Department of Biochemistry, Institute for Protein Design, University of Washington, Seattle, 98105 WA USA

**Keywords:** Biocatalysis, Enzymes, Metabolic engineering, Biocatalysis

## Abstract

Prenylation of natural compounds adds structural diversity, alters biological activity, and enhances therapeutic potential. Because prenylated compounds often have a low natural abundance, alternative production methods are needed. Metabolic engineering enables natural product biosynthesis from inexpensive biomass, but is limited by the complexity of secondary metabolite pathways, intermediate and product toxicities, and substrate accessibility. Alternatively, enzyme catalyzed prenyl transfer provides excellent regio- and stereo-specificity, but requires expensive isoprenyl pyrophosphate substrates. Here we develop a flexible cell-free enzymatic prenylating system that generates isoprenyl pyrophosphate substrates from glucose to prenylate an array of natural products. The system provides an efficient route to cannabinoid precursors cannabigerolic acid (CBGA) and cannabigerovarinic acid (CBGVA) at >1 g/L, and a single enzymatic step converts the precursors into cannabidiolic acid (CBDA) and cannabidivarinic acid (CBDVA). Cell-free methods may provide a powerful alternative to metabolic engineering for chemicals that are hard to produce in living organisms.

## Abstract (web summary)

Producing individual cannabinoids by metabolically engineered microbes has proven challenging. Here, the authors develop a cell-free enzymatic prenylating system to generate isoprenyl pyrophosphate substrates directly from glucose and produce both common and rare cannabinoids at >1 g/L.

## Introduction

**Fig. 1:**
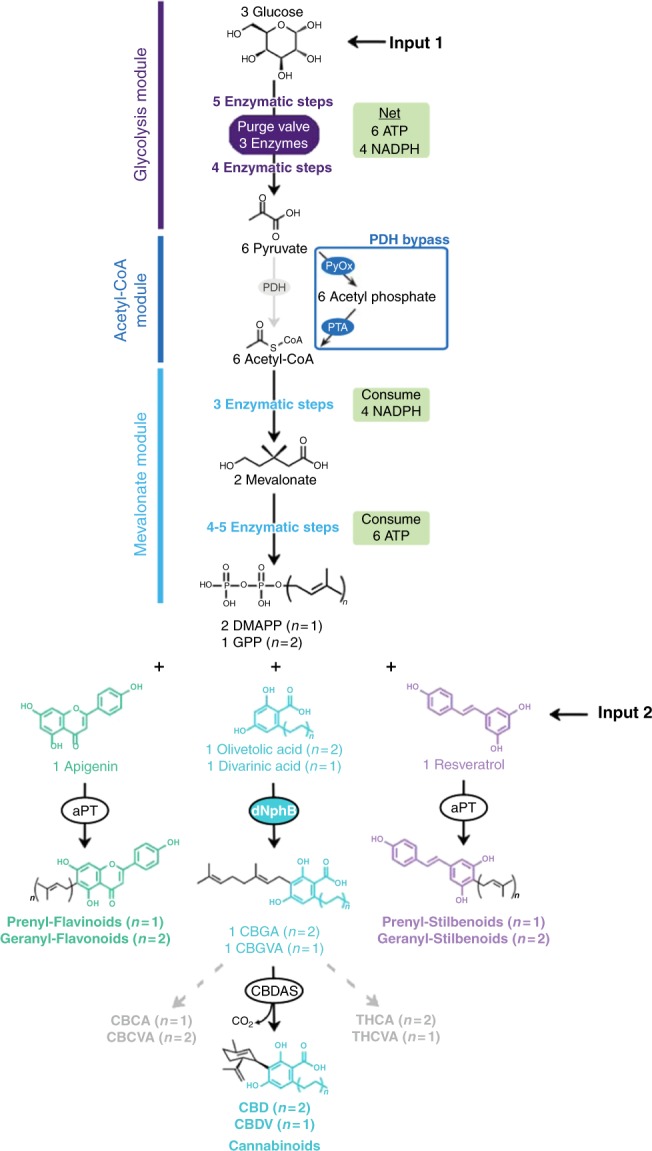
The synthetic biochemistry platform for the production of prenyl-natural products. First, glucose is broken down into pyruvate through a glycolysis pathway modified to regulate NADPH levels (22) (12 enzymatic steps). Then, either PDH or the PDH bypass converts pyruvate into acetyl-CoA. Acetyl-CoA is converted into GPP via the mevalonate pathway (eight enzymatic steps). By varying the aromatic prenyltransferase (aPT) and aromatic substrate we are able to produce various prenyl-flavonoids and prenyl-stilbenoids using the same central pathway. We developed variants of the prenyltransferase NphB (dNphB) to produce CBGA or CBGVA. CBGA is converted to cannabidiol and CBGVA is converted to cannabidivaric acid via cannabidiolic acid synthase (CBDAS). It is possible to produce other cannabinoids by using different cannabinoid synthases (THCAS and CBCAS)

Prenylated natural products are a large class of bioactive molecules with demonstrated medicinal properties^1^. Examples include prenyl-flavanoids, prenyl-stilbenoids, and cannabinoids (see Fig. 1). Cannabinoids in particular show immense therapeutic potential with over 100 ongoing clinical trials as antiemetics, anticonvulsants, antidepressants, and analgesics^2–6^. Nevertheless, despite the therapeutic potential of prenyl-natural products, their study and use is limited by the lack of cost-effective production methods. Plant-derived prenyl-compounds are difficult to isolate due to the structural similarity of contaminating molecules, and the variable composition between crops^7,8^. These challenges are further exacerbated when attempting to isolate low abundance compounds. Many chemical syntheses have been developed to address the challenges associated with making prenylated natural products, but they are generally impractical for drug manufacturing due to the degree of complexity and low yields^9–11^.

Microbial production is a useful alternative to natural extraction for prenylated natural products, but comes with many challenges such as the need to divert carbon flux from central metabolism and product toxicity to name a few. For example, prenyl-natural products like prenyl-naringenin, prenyl-resveratrol, and cannabidiol (CBD) are derived from a combination of the metabolic pathways for fatty acid, isoprenoid, and polyketide biosynthesis. So, high-level production requires efficient re-routing of long, essential and highly regulated pathways. Despite the challenges, many groups have engineered microbes to produce unprenylated polyketides, like naringenin, resveratrol, and olivetolate, but at relatively low levels (110, 391, and 80 mg/L, respectively)^12,13^. Obtaining prenylated products is even more challenging because geranyl-pyrophosphate (GPP) is an essential metabolite that is toxic to cells at moderate concentrations, creating a significant barrier for high-level microbial production^14^. So, in spite of intense interest, to our knowledge there are no published reports of the complete biosynthesis of prenyl-flavonoids, prenyl-stilbenoids, or cannabinoids in recombinant microbes.

Much recent effort has focused on alternative methods for cannabinoid production. Two groups have produced the polyketide cannabinoid intermediate, olivetolic acid (OA) at low levels in yeast (0.5 mg/L) or *E. coli* (80 mg/L), but did not prenylate OA or produce a cannabinoid from the biosynthesized OA^13,15^. In other work, tetrahydrocannabinolic acid was produced in cell extracts from either exogenously added GPP and OA in a two enzyme pathway^16^ or from cannabigerolic acid (CBGA) using a single enzyme^17^. However, it is unclear how GPP or CBGA could be obtained at sufficient levels for economical production due to the high cost of these molecules.

Here, we propose an alternative biological approach to prenylated natural product biosynthesis using a cell-free enzymatic platform we call synthetic biochemistry, which has shown great promise for the production of bio-based molecules^18–24^. The synthetic biochemistry approach frees us from worrying about the toxicity of products and intermediates, affords rapid design-build-test cycles, precise control of all system components, and complete flexibility in pathway design. Nevertheless, building highly complex systems involving dozens of enzymes, associated cofactors and myriad metabolites on a large scale outside the context of the cell is an enormous challenge. One of the keys to making commercially viable cell-free systems is reducing enzyme costs by employing stable enzymes that can last for long periods of time. Recently, Zhang and co-workers converted maltodextrin into inositol at a 20,000 L scale in a five enzyme system using thermophilic enzymes purified by simple heating step^25^, demonstrating that at least simple cell-free systems can reach industrial scale. Another key requirement is designing systems that effectively generate and recycle high energy cofactors (ATP, NAD(P)H) so that they can be used many times. We have previously reported a flexible enzymatic purge valve and rheostat for regulating the supply of reducing equivalents and ATP^26–28^, allowing us to build systems that run for many days and produce high titers of isobutanol and terpenes. Here, we employ these concepts to develop cell-free production of a variety of prenylated compounds. We use glucose as a feedstock to produce GPP and optimize the system for the high-titer production of the cannabinoid compounds CBGA and cannabigerovarinic acid (CBGVA).

## Results

### Construction of the cell-free prenylation pathway

Our synthetic biochemistry approach is outlined in Fig. 1 (detailed in Supplementary Figure 1) and expands on a system we developed previously for terpene production^26^. First, glucose is broken down via a modified glycolysis pathway to produce high-energy cofactors ATP and NADPH in addition to the carbon building block, acetyl-CoA using an alternative pyruvate oxidation pathway^26^. The acetyl-CoA is then assembled into the prenyl-donor compound, GPP, via the mevalonate pathway using the ATP and NADPH produced from glycolysis. Importantly, a purge valve^26^ introduced into the glycolysis pathway balances NADPH production and consumption while maintaining carbon flux. The prenylation module then uses the GPP to prenylate exogenously added substrate to yield the desired prenylated product. To expand the capabilities of our synthetic biochemistry platform we developed a prenylating system that employs a nonspecific prenylating enzyme such as NphB, AtaPT, or NovQ to produce an array of prenyl-compounds derived from glucose^29–31^. We then further engineered NphB using Rosetta to specifically prenylate OA.

**Fig. 2:**
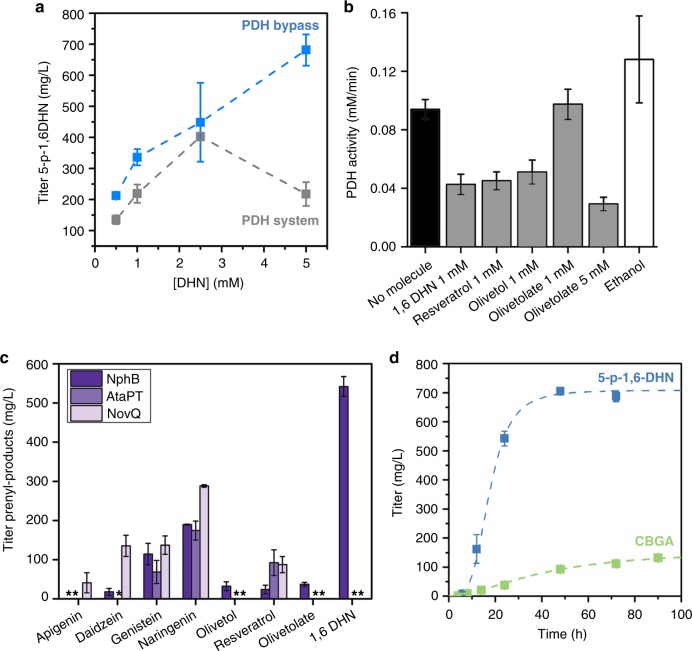
Development of a PDH bypass for the prenylation of aromatic polyketides. **a** The comparison of the final titers achieved with the full pathway utilizing PDH (PDH system-gray trace) and the PDH bypass system (blue trace) at different concentrations of 1,6 DHN. (biological replicates, *n* = 3). **b** The activity of pyruvate dehydrogenase (*E. coli* PDH) measured in the presence of various aromatic polyketides and 2% ethanol (vehicle) (biological replicates, *n* = 3). **c** Various aromatic substrates were added to the pathway with either NphB, AtaPT, or NovQ prenyltransferase (biological replicates, *n* = 3). The result is a variety of C5 and C10 prenyl-natural products. (* Indicates titer not determined). **d** Production of 5-prenyl-1,6 DHN (blue trace) over time compared to a separate reaction to produce CBGA (green trace). Both reactions utilized the PDH bypass and WT NphB (biological replicates, *n* = 3). All error ranges reflect the standard deviation. Source data for Fig. 2a–d are provided as a Source Data file

As a first test of the system, we built the full cell-free system (23 enzymes) to generate GPP from glucose and employed wild-type NphB to prenylate its preferred substrate 1,6 dihydroxynapthalene (1,6 DHN; added exogenously). 1,6 DHN was added at the beginning of the reaction along with glucose. Up to ~400 mg/L (1.3 mM) of prenylated product was obtained from 2.5 mM 1,6 DHN. However, increasing the 1,6 DHN concentration from 2.5 to 5 mM, decreased final titers ~2-fold suggesting that 1,6 DHN inhibited one or more enzymes (Fig. 2a). Enzyme assays revealed that pyruvate dehydrogenase (PDH) was inhibited by 1,6 DHN, as well as olivetol, resveratrol, and olivetolate (Fig. 2b). Therefore, to engineer a general prenylation system, we sought to eliminate PDH.

To remove the need for PDH, we implemented a PDH bypass (Fig. 1). In the PDH bypass, pyruvate is converted to acetyl-CoA using a pyruvate oxidase (PyOx) to produce acetyl-phosphate followed by the action of acetyl-phosphate transferase (PTA). The PDH bypass has two advantages. First, PDH is a large enzyme complex that is difficult to work with, so bypassing PDH streamlines enzyme production. More importantly, initial experiments revealed that the bypass is not subject to the inhibition seen at higher concentrations of 1,6 DHN. Once we confirmed the PDH bypass improved 1,6 DHN titers, we began to optimize the system as a general prenylation system. We varied co-factor concentrations, protein levels, and environmental conditions such as temperature and pH to identify the ideal set of conditions. Throughout this process we found that ATP, NADP^+^, phosphate, and NphB concentrations had the greatest impact on the final titer. As shown in Fig. 2a, when we employed the PDH bypass, we found a 4-fold increase in titers of 5-prenyl-1,6 DHN when starting with 5 mM 1,6 DHN (Fig. 2a). When utilizing the PDH bypass system, approximately 50% of 1,6 DHN was converted in 24 h, reaching a final titer of 705 ± 12 mg/L (all error ranges in this work reflect the standard deviation) (Fig. 2b).

We then tested the ability of the PDH bypass cell-free system to prenylate a variety of aromatic substrates (apigenin, diadzein, genestein, naringenin, olivetol, OA, and resveratrol). All the aromatic substrates were prenylated using one or more of the tested prenyl transferases (Fig. 2c and Supplementary Figures 5–10). Thus, it is possible to produce a variety of prenylated natural products using a cell-free enzymatic system to generate the expensive co-substrates GPP and DMAPP. Further, the ease with which an exogenous substrate can be added to a synthetic biochemistry system is a great advantage because it is often not possible to add co-substrates exogenously to microbes since they cannot enter the cell^16^.

To test whether we could use synthetic biochemistry to produce high levels of therapeutically relevant prenylated products, we focused optimization efforts on cannabinoids due to the growing interest in new ways to make these medically important compounds. As shown in Fig. 2d the initial system produced the cannabinoid precursor CBGA at a constant rate of 2.1 mg L^−1^ h^−1^ over 72 h and reached a final titer of only 132 mg/L (Fig. 2d).

### Redesign of NphB to improve CBGA synthesis

Although the system produced CBGA, there were two problems. First, the turnover rate of the prenyltransferase NphB for CBGA production is extremely poor (*k*_cat_ = 0.0021 ± 0.00008 min^−^^1^, Supplementary Table 5). Second, prenylation of OA by NphB is highly nonspecific, generating a major side-product, 2-O-geranyl olivetolate^16^. We therefore, sought to improve CBGA production by enhancing the activity and specificity of NphB by design.

**Fig. 3:**
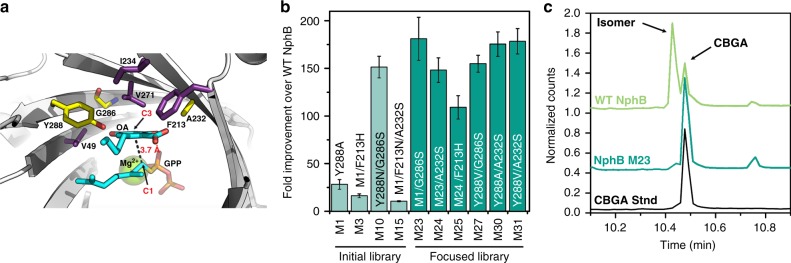
Engineering NphB to improve CBGA production. **a** A model of olivetolate in the active site of WT NphB (1ZB6). Residues highlighted in yellow and purple were allowed to vary during the design process. The residues in yellow had the largest effects on activity with OA and were the positions targeted in the focused library. **b** The results of an activity assay to determine the approximate activity of NphB mutants with olivetolate as the substrate. The fold improvement is an average of triplicate reactions with GPP (2.5 mM), olivetolate (5 mM), MgCl_2_(5 mM), and 1 mg/mL of WT or mutant NphB (biological replicates, *n* = 3, error range is standard deviation). **c** GC–MS chromatograms of the full pathway reaction products using M23 or WT NphB compared to a CBGA standard. Source data for Fig. 3b, c are provided as a Source Data file

Briefly, OA was docked into the active site of the NphB crystal structure (Fig. 3a)^32^, then Rosetta was used to predict mutations that would improve OA binding. We narrowed the Rosetta results to a 22 construct library (see Supplementary Tables 2 and 3), and screened for CBGA production (Supplementary Table 4). We made several key observations during the initial screen, shown in Supplementary Figure 2: (1) Y288A (M1) and Y288N (M2) by themselves dramatically enhanced activity, as predicted by computation; (2) the presence of Y288N in any construct decreased the enzyme yield suggesting Y288N may be a destabilizing mutation (making Y288A the preferred mutation); (3) the addition of G286S in the Y288N (M10) background appeared to improve activity further over Y288N (M2), suggesting that G286S could be another favorable mutation; (4) we noted an activity improvement of Y288A/F213N/A232S (M15) over Y288A/F213N (M5) suggesting that A232S may also be a favorable mutation. From these initial observations we constructed a focused library with all but one of the constructs in the second library exhibiting activity at least 100-fold higher than WT NphB in an endpoint assay (Fig. 3b).

The best two constructs, M23 and M31, exhibited dramatically improved activity and specificity. Both had *k*_cat_ values 1000-fold higher than WT NphB and both produce only the correct prenylated isomer, CBGA. As shown in Fig. 3c, WT NphB produces CBGA, but the dominant product is a prenylated side-product, 2-O-geranyl olivetolate, whereas M23 makes CBGA almost exclusively. Overall, the designed enzyme is a much more active and specific CBGA synthase than WT NphB, and is easier to work with than the natural cannabis prenyltransferase, which is an integral membrane protein^16,33^. Our soluble, CBGA synthase (M23) could potentially be applied in both cell-free and in vivo systems to improve cannabinoid production.

### Improved production of cannabinoids

**Fig. 4:**
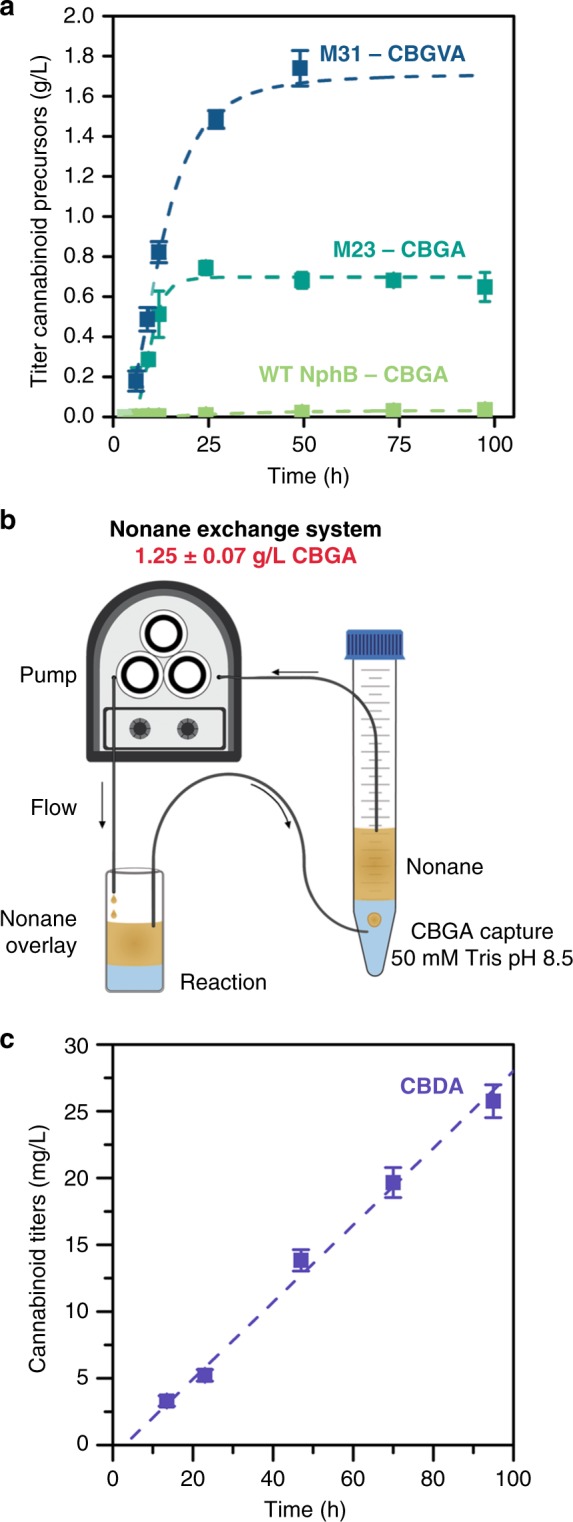
Evaluation of the cell-free prenylation system for the production of various cannabinoids. **a** The cell-free enzymatic production (from glucose) of cannabinoid precursors over time. CBGA production using M23 is shown in the dark aqua trace and WT NphB in the light green trace. The production of CBGVA using M31 is shown in the dark blue trace. The concentration of WT, M23, and M31 NphB was fixed at 0.5 mg/mL (biological replicates, *n* = 3). **b** Using a nonane-flow CBGA capture system, we were able to obtain a higher titer of CBGA (1.2 g/L). The nonane layer was exchanged using a peristaltic pump, which circulated the nonane in the direction indicated by the arrows. **c** Production of CBDA over time using CBDAS (biological replicates, *n* = 3). All error ranges are the standard deviation. Source data for Fig. 4a, c are provided as a Source Data file

With our designed CBGA synthase in hand (M23), we tested the ability to produce CBGA directly from glucose and OA using the full synthetic biochemistry system, including the PDH bypass (Fig. 1). The initial productivity of the system using M23 was 67 mg L^−1^ h^−1^ with a final titer of 744 ± 34 mg/L CBGA—100-fold faster and 21-fold higher titer than CBGA production using WT NphB (Fig. 4a). We noted that with the mutant NphB enzyme, maximum titers were reached within 24 h, after which production spontaneously stopped. In contrast, the system with the wild-type enzyme ran continuously for up to 4 days, suggesting enzymes and cofactors remain active and viable for longer periods of time, consistent with prior work^26^. So what is stopping the reaction at the higher titers? We observed that reactions turned cloudy once ~500 mg/L CBGA was produced. We collected the precipitate and identified a mix of enzymes in the precipitate by sodium dodecyl sulfate polyacrylamide gel electrophoresis (SDS-PAGE) analysis (Supplementary Figure 3), indicating high-levels of CBGA in solution may be causing enzymes to precipitate. We, therefore, sought to continually remove the product in situ during the reaction (a capability difficult to implement in living systems).

Initially a fixed volume nonane overlay was used for each reaction to extract CBGA. Unfortunately, CBGA is more soluble in water than nonane, limiting the amount of CBGA that can be extracted with a simple overlay. We therefore designed a flow system that would capture CBGA from the nonane layer and trap it in a separate buffered reservoir (Fig. 4b). By implementing this flow system we hoped to maintain a lower concentration of CBGA in the reaction vessel to mitigate enzyme precipitation. The flow system indeed improved the final titers to 1.25 ± 0.07 g/L, however, enzyme precipitation still occurred at about 24 h.

We next evaluated the system flexibility by replacing OA with divarinic acid (DA) to produce the precursor of many rare cannabinoids, CBGVA. We first tested whether our designed enzymes would be active with DA as the substrate. Kinetic analysis (Table S5) indicated that M31 effectively prenylates DA, with catalytic efficiencies 15-fold higher than M23 and 650-fold higher than WT NphB. We, therefore, utilized M31 to produce CBGVA from glucose and DA. As shown in Fig. 4a, CBGVA was produced at a maximum productivity of ~107 mg L^−1^ h^−1^, and reached a final titer of 1.74 ± 0.09 g/L, converting 92% of the added DA to CBGVA. The nonane-flow system was not needed for the production of CBGVA because CBGVA was less potent in precipitating enzymes.

### Production of additional cannabinoids

To illustrate the production of other cannabinoids from the central cannabinoids CBGA and CBGVA, we employed CBDA synthase to convert CBGA into CBDA and CBGVA into CBDVA. Conversion of CBGA into CBDA has been demonstrated by several groups^17,34–36^. In our case, we simply transferred the nonane overlay containing CBGA to an aqueous solution containing CBDA synthase, and indeed we were able to convert CBGA into CBDA at a constant rate of 14.4 ± 0.8 mg L^−1^ h^−1^ mg total protein^−1^ over the course of 4 days converting 25% of the CBGA added to CBDA (Fig. 4c). To our knowledge it is not known whether CBGVA can be converted into the rare cannabinoid CBDVA using the CBDA synthase. So we added CBGVA, extracted from the cell-free system, to a reaction containing CBDA synthase. CBDVA was produced (Supplementary Figure 4) by CBDA synthase at a rate of 7.1 ± 0.1 mg L^−1^ h^−1^ mg total protein^−1^ for 24 h. We note that the cannabinoid acids can undergo spontaneous decarboxylation or heat induced decarboxylation to ultimately form additional bioactive cannabinoids CBD and cannabidivarin (CBDV). Thus, our system provides opportunities for ultimately producing a wide-variety of cannabinoids.

## Discussion

Our results demonstrate the power and flexibility of a cell-free approach, not only for the production of pure, therapeutically relevant cannabinoids and other prenylated natural products, but for bio-derived chemicals in general. Freedom from worries about cell viability allowed us to focus on pathway optimization rather than minimizing GPP toxicity, while the lack of a cell membrane barrier freed us to design a system with added aromatic molecules, which would not be possible in cells. Moreover, we could flexibly change the input from OA to DA to target rare cannabinoids without redesigning an entire pathway. Finally, it was straightforward to identify and focus our efforts on fixing the bottleneck steps. When we started this project we were only able to produce 9 mg/L of CBGA using the monoterpene pathway developed by Korman et al.^26^. By introducing the PDH bypass and optimizing for cofactors, enzymes and environmental factors we were able to increase those titers to 132 mg/L. To improve titers further we engineered the NphB prenyltransferase, which further increased titers to 600 mg/L of CBGA. The final bottleneck was enzyme stability in the presence of CBGA, so by limiting the CBGA in the reaction vessel, we increased the titer to 1.25 g/L of CBGA, nearly a 140-fold improvement. Solutions were quickly implemented due to speedy design-build-test cycles, rapidly yielding results that far exceed published results using living cells. Like all new technology, the current system will need additional technical developments to become commercially viable, but our results suggest that synthetic biochemistry can become a realistic option for producing bio-based chemicals.

## Methods

### Chemicals and reagents

Yeast hexokinase and *Corynebacterium glutamicum* catalase were purchased from Sigma Aldrich. *Aerococcus viridians* pyruvate oxidase was purchased from A.G. scientific. All cofactors and reagents were purchased from either Sigma Aldrich or Thermo Fisher Scientific, with the exception of OA, which was purchased from Santa Cruz Biotechnology and divarinic acid, which was purchased from Toronto Research Chemicals.

### Cloning and purification of enzymes

The NphB gene was purchased as a gene block from IDT DNA, and cloned into a pET 28(+) vector using the Gibson Assembly method. The gene block sequences are provided in Supplementary Data 1. The remaining enzymes were amplified from genomic DNA or a plasmid, and cloned into pET28(+) using the same Gibson assembly method. The primer sequences used for amplification in this work are listed in Supplementary Table 6. All plasmids were transformed into BL21(DE3) Gold, and enzymes expressed in LB media with 50 µg/mL kanamycin. One litre cultures were inoculated with 2 mL of a saturated culture in the same media, and grown to an OD_600_ of 0.5–0.8 at 37 °C. The cultures were induced with 1 mM IPTG, and expressed at 18 °C for 16 h. The cells were harvested by centrifugation at 2500×*g*, and resuspended in ~20 mL lysis buffer: 50 mM Tris [pH 8.0], 150 mM NaCl, and 10 mM imidazole. The cells were lysed using an Emulsiflex instrument. The lysate was clarified by centrifugation at 20,000×*g*, and the supernatant was batch bound to 1 mL NiNTA resin for 30 min at 4 °C. The resin was transferred to a gravity flow column. The resin was washed with 10 column volumes of wash buffer: 50 mM Tris [pH 8.0], 150 mM NaCl, and 10 mM imidazole. The protein was then eluted with 2 column volumes of elution buffer: 50 mM Tris [pH 8.0], 150 mM NaCl, 250 mM imidazole, and 30% (v/v) glycerol. Enzymes were flash frozen in elution buffer using liquid N_2_, and the enzyme stocks were stored at −80 °C.

### PDH cell-free reactions

The PDH reactions were assembled in two parts. First the cofactors and substrates were combined in one tube, and the enzymes were combined in another. The reactions were initiated by mixing the cofactors and enzymes in a final volume of 200 µL. The final substrate and co-factor concentrations were as follows: 500 mM glucose, 1 mM 1,6 fructose bisphosphate, 4 mM ATP, 0.5 mM 2,3 bisphosphoglycerate, 0.5 mM NAD^+^, 1.5 mM CoA, 1.5 mM NADP^+^, 0.5 mM TPP, 6 mM MgCl_2_, 10 mM KCl, 50 mM Tris [pH 8.0] and 20 mM phosphate buffer [pH 8.0], 5 mM glutathione, and 0.5–5 mM 1,6 DHN. The enzyme amounts added to the reaction can be found in Supplementary Table 1 (PyOx and PTA were not added to these reactions). The reactions were quenched at 24 h.

### PDH activity assays

PDH was assayed for activity in the presence of several aromatic polyketides. The vehicle control was 1% ethanol, and the activity was compared to an assay without the aromatic polyketides. The final reaction volume was 200 µL, and contained 2 mM NAD^+^, 2 mM CoA, 1 mM TPP, 5 mM MgCl_2_, 5 mM KCl, 50 mM Tris pH 8.0, and 5 µL of 1.25 mg/mL PDH. The reactions were set up in a 96-well plate. The aromatic polyketides (dissolved in ethanol) were added to a final concentration of 1 mM and the ethanol control was added to a final concentration of 1% (v/v). The plate was incubated at room temperature for 10 min, and the reactions were initiated with 10 µL of 100 mM pyruvate. The absorbance at 340 nm was monitored for 10 min using an M200 spectrometer. Because the aromatic molecules had a background absorbance at 340 nm, the reactions were blanked using the reaction mixture and aromatic molecule, but instead of initiating the reaction with pyruvate, water was added. The initial rates were determined using the initial slope of a linear fit. The amount of NADH produced per unit time was calculated using Beer’s law, and the extinction coefficient of 6.22 × 10^3^ M^−1^ cm^−1^. Reactions were performed in triplicate, and the average value and standard error were calculated.

### PyOx/PTA cell-free reactions

The PyOx/PTA reactions were assembled in two pieces. First the cofactors and substrates were combined in one tube, and the enzymes were combined in another. The final co-factor and substrate concentrations in the 200 µL reaction were as follows: 500 mM glucose, 1 mM 1,6 fructose bisphosphate, 4 mM ATP, 0.5 mM 2,3 bisphosphoglycerate, 0.5 mM NAD^+^, 1.5 mM CoA, 3 mM mM NADP^+^, 0.5 mM TPP, 6 mM MgCl_2_, 10 mM KCl, 50 mM Tris, and 50 mM phosphate buffer [pH 8.0]. The amount of enzyme added to each reaction is detailed in Supplemental Table 1. The cofactors and enzymes were mixed to initiate the reaction, and a 500 µL nonane overlay was added to the top. The reactions were incubated at room temperature shaking gently on a gel shaker.

When the aromatic substrate was the varied component 0.5–5 mM of the aromatic substrate was added to the reaction, and the reactions were quenched at 24 h. When time was the varied component, 5 mM of 1,6 DHN was added, and separate reactions were quenched at ~12, 24, 48, and 72 h.

Conditions for the olivetolate and divarinic acid reactions (produced CBGA and CBGVA, respectively) were altered slightly. Optimization of the cannabinoid pathway showed that the same titers could be achieved with less glucose, so we reduced the glucose concentration to 150 mM (we did not test lower glucose concentrations). Additionally, increasing the NADP^+^ concentration to 6 mM and decreasing the ATP concentration to 1 mM led to higher titers of CBGA. The olivetolate concentration was set at 5 mM. The amount of NphB added to the reaction was variable. The data shown in Fig. 2c utilized 1.5 mg/mL NphB, and the reactions were quenched at ~4, 8, 14, 24, 48, 72, and 96 h. The data shown in Fig. 4a was achieved with 0.5 mg/mL of WT NphB and M23 and M31 (for divarinic acid), and reactions were quenched at ~6, 9, 12, 24, 48, 72, and 96 h.

The conditions were identical to the method above with the following exceptions, the final concentration of the aromatic substrates was 1 mM and the initial glucose concentration was 150 mM. Additionally, the final concentration of the prenyl-transferase was 1 mg/mL, and we tested AtaPT, NovQ, and NphB with apigenin, daidzein, genistein, naringenin, and resveratrol. We also tested NphB with olivetol, olivetolate, and 1,6 DHN. The reactions were quenched at 24 h.

### Quenching reactions

To quench the reactions, the aqueous and organic layer were transferred to a 1.5 mL microcentrifuge tube. The reaction vial was washed with 200 µL of ethyl acetate, which was then pooled with the reaction in the microcentrifuge tube. The samples were vortexed for 5–10 s and then centrifuged for 3 min at 16,060 × *g*. The organic layer was removed, and the remaining aqueous layer was extracted two additional times with 200 µL of ethyl acetate. For each sample the organic extract was pooled, and then evaporated using a vacuum centrifuge. The samples were redissolved in methanol for HPLC analysis. Due to the observed protein precipitation, the CBGA reactions shown in Fig. 4a were extracted in the presence of 0.12 g of urea (solid), to facilitate the extraction of CBGA. This was unnecessary for the WT NphB CBGA data in Fig. 2c because the proteins did not precipitate.

### Quantification of products

The reactions were fractionated by reverse phase chromatography on a C18 column (4.6 × 100 mm) using a Thermo Ultimate 3000 HPLC. The column compartment temperature was set to 40 °C, and the flow rate was 1 mL/min. The compounds were separated using a gradient elution with water +0.1% TFA (solvent A) and acetonitrile +0.1% TFA (solvent B) as the mobile phase. Solvent B was held at 20% for the first min. Then solvent B was increased to 95% B over 4 min, and 95% B was then held for 3 min. The column was then re-equilibrated to 20% B for three min, for a total run time of 11 min.

The cannabinoids (CBGA, CBDA, and CBDVA) were quantified using an external calibration curve derived from an analytical standard purchased from Sigma Aldrich. The 5-p-1,6-DHN and CBGVA nuclear magnetic resonance (NMR) samples were used to generate an external calibration curve because authentic standards were not available (see below). A known concentration of the standard was dissolved in water, and then extracted using the method detailed above.

### Quantify prenyl-products without authentic standards

Due to the lack of authentic standards for the prenyl-products prenyl-apigenin, prenyl-daidzein, prenyl-naringenin, prenyl-genistein, prenyl-resveratrol, and prenyl-olivetol, we quantified the prenyl-products based on substrate consumption. To generate a standard curve, serial dilutions of each aromatic substrate were subjected to the reaction mix, but to prevent product formation the prenyl-transferase was left out. We used liquid chromatography–mass spectrometry to quantify the amount of substrate consumed by the reaction compared to the standard curve.

Electrospray ionisation time-of-flight measurements were carried out on a Waters LCT-Premier XE Time of Flight Instrument controlled by MassLynx 4.1 software (Waters Corporation, Milford, MA). The instrument was equipped with the Multi Mode Ionization source operated in the electrospray mode. A solution of Leucine Enkephalin (Sigma Chemical, L9133) was used in the Lock-Spray to obtain accurate mass measurements. Samples were infused using direct loop injection on a Waters Acquity UPLC system. Samples were separated on a Waters Acquity UPLC system using an Acquity BEH C18 1.7 µm column (50 × 2.1 mm) and were eluted with a gradient of 30–95% solvent B over 10 min (solvent A: water, solvent B: acetonitrile, both with 0.2% formic acid (vol/vol)). Mass spectra were recorded from a mass of 300–2000 Da.

### NMR spectroscopy

NMR spectroscopy was used to identify prenyl-products, and quantify 5-p-1,6-DHN. The PyOx/PTA cell-free system was used to produce prenyl-DHN. A total of 200 µL reactions were pooled, and extracted three times with an equivalent amount of nonane and then the nonane was evaporated. The product of the reactions was suspended in 500 µL of deuterated methanol (CD_3_OD), with 2 mM 1,3,5-trimethoxybenzene (TMB) as an internal standard. Spectra were collected on an AV400 Bruker NMR spectrometer. The amount of the prenylated compound in the sample was determined with reference to the internal TMB standard. We compared the proton signal from TMB (3H, singlet) at 6.05 ppm with an aromatic proton corresponding to 5-p-1,6-DHN (1H, doublet) at 7.27 ppm.

NMR was also used to identify the product of the enzymatic system with divarinic acid as the aromatic substrate. The PyOx/PTA system was set up as detailed above, and the reactions were quenched at 24 h. The reactions were extracted as detailed above (under the subheading: Quenching Reactions), and analyzed on the HPLC. There was a new major peak at 6.7 min that we predicted to be the prenylated divarinic acid. We HPLC purified the peak, removed the solvent, and redissolved the pure component in 600 µL of CD_3_OD. A proton spectrum collected with an AV500 Bruker NMR spectrometer was compared to a proton spectrum published by Shoyama et al.^37^ for CBGVA to confirm that CBGVA was the main product. Based on the report by Shoyama et al. the study by Bohlmann et al.^38^, we conclude that the prenylation of divarinic acid occurs at the C3 carbon of divarinic acid. Shoyoma et al.^37^ published the chemical shifts of CBGVA in CD_3_OD, so by direct comparison of our NMR spectra to the published chemical shifts we conclude that we produced CBGVA. This is further supported by the work conducted by Bohlmann, which suggests that if the prenylation occurred at the C5 site, we would observe a proton with a chemical shift around 5.8 ppm, which we did not observe, Supplementary Figure 11.

### Rosetta design to modify the binding pocket of NphB

We placed olivetolate in the active site of NphB in six different starting positions denoted as Olivetolate P1–6 in Table S2. We ran ROSETTA 5 times for each olivetolate position for a total of 30 designs. The mutations predicted in each design are listed in Table S2. For each olivetolate position we chose a consensus set of mutations (i.e., the most frequently chosen residue) to evaluate further: Consensus Group A through F (Table S2). We then sought to evaluate the relative importance of each ROSSETTA suggested mutation. For each Consensus Group, we set the mutations back to WT residue, one at a time, and used ROSETTA to calculate the change in energy score (see Table S3). Those that caused the largest change in energy were deemed to be the most important mutants to include in the library for experimental testing.

To model the OA, we took the 4MX.sdf 3-D structure of olivetolate from the 5B09 crystal structure and added hydrogen atoms to the structure assuming pH 7 using Open Babel 2.3.1^39^. A rotamer library was generated for OA using the Bio Chemical Library (BCL) molecule: Conformer Generator 3.5 using the PDB library^40^. Finally, the aromatic bonds were manually annotated into the file before generating the parameter file read by Rosetta using the script main/source/python/public/molfile_to_params.py in the Rosetta 3.7 release. The parameter file for geranyl s-thioldiphosphate (GST) was generated without a rotamer library using the GST.sdf file from the 1ZB6 crystal structure. The OA molecule was then manually placed into the co-crystal structure of NphB with GST and DHN (1ZB6) with the DHN and crystallographic waters removed using pymol. The OA was placed in 6 different positions in the active site with the plane of the olivetolate aromatic ring parallel to the GST alkyl tail and the desired prenylation site 3.7 angstroms away from the eventual carbocation mirroring the placement of DHN in the 1ZB6 crystal structure. Residues 49, 162, 213, 224, 232, 233, 234, 271, 286, and 288 were allowed to be any amino acid during the Rosetta design with other sidechains held in a fixed position and the backbone fixed. The designed residues were in direct contact with the olivetolate and not in direct contact with GST. The fixed backbone script main/source/bin/fixbb.static.linuxgccrelease from the Rosetta 3.7 release was run with the all possible rotamers (-ex4), using the input sidechains (-use_input_sc), sidechains minimized after design (minimize_sidechains), the linear memnode interaction graph (-linmem_ig 10), and both with and without the ligand weighted score function (-score:weights ligand). From the identical starting point each design was run five times using the -nstruct input.

### Initial NphB mutant library screening

For screening of the initial library, we performed small scale expression and purifications. In total, 25 mL of LB media with 50 µg/mL of kanamycin was inoculated with 25 µL of a saturated culture of BL21 DE3 Gold harboring the NphB expression plasmid. The cultures were incubated at 37 °C until the OD_600_ reached 0.4–0.6. The expression of the NphB constructs were induced with the addition of 1 mM IPTG, followed by incubation for 18 h at 18 °C. Cells were harvested by centrifugation at 2500 × *g*. The pellets were resuspended in 500 µL of lysis buffer: 50 mM [Tris pH 8.0], 150 mM NaCl, and 5 mM imidazole and lysed by sonication. The cell lysate was clarified by centrifugation at 20,000 × *g* for 10 min at 4 °C, and the supernatant was incubated at 4 °C with 50 µL of NiNTA resin. A 96-well spin column plate was used to purify the NphB constructs. The supernatant/resin was applied to the column and centrifuged for 2 min at 500 × *g*. A total of 500 µL of lysis buffer was then added, and the plate was centrifuged again for 1 min at 500 × *g*. The protein was eluted using 200 µL of elution buffer (50 mM Tris [pH 8.0], 150 mM NaCl, 250 mM imidazole and 30% (v/v) glycerol).

The enzymes were assayed under the following conditions: 2.5 mM geranyl-pyrophosphate, 5 mM olivetolate, 5 mM MgCl_2_, 50 mM Tris pH 8.0, ~0.1 mg/mL NphB mutant in a final volume of 100 µL. All enzymes were first diluted to 0.5 mg/mL using elution buffer so the final concentration of imidazole was the same in each reaction. The reactions were incubated for 12 h at room temperature, then extracted 3 times with 100 µL of ethyl acetate. The organic extract was pooled for each reaction and the solvent was removed using a vacuum centrifuge. The samples were redissolved in 100 µL of methanol and subjected to HPLC analysis.

### Focused NphB mutant library screening

For the focused library, we performed 1 L scale expression and purification of the NphB constructs as described above. The enzymes were assayed under the following conditions: 2.5 mM GPP, 5 mM olivetolate, 5 mM MgCl_2_, 50 mM Tris pH 8.0, and ~1 mg/mL of NphB enzyme in a final volume of 100 µL. The reactions were incubated at room temperature for 1 h. A total of 40 µL of each reaction was quenched in 80 µL of acetonitrile. The samples were centrifuged for 5 min at 16,060 × *g*, to remove precipitated proteins. The supernatant was analyzed using HPLC as described above.

### Enzyme kinetic parameters

The reactions were set up under the following conditions: 50 mM Tris [pH 8.0], 2.5 mM GPP, 5 mM MgCl_2_, ~27 µM enzyme, and olivetolate or divarinic acid was varied from 0.1 to 6 mM in a final volume of 200 µL. A total of 40 µL of the reaction was quenched in 80 µl acetonitrile +0.1% TFA, at the time intervals detailed below. The reactions were centrifuged for 5 min at 13,000–16,060 × *g* to pellet the protein, and the supernatant was analyzed using the HPLC method detailed above. The initial rate was plotted vs the concentration of substrate, and fit with the Michaelis–Menten equation to determine the kinetic parameters *k*_*cat*_ and *K*_M_ (OriginPro). Each Michaelis–Menten curve was performed in triplicate. The average and standard deviation of the kinetic parameters are reported. The time courses with olivetolate as the substrate were as follows: for WT, M1, M10, and M30 the time course was 3, 6, 9, and 12 min. For M25 the reactions were quenched at 1, 2, 4, and 8 min, and for M31 the reactions were quenched at 1, 2, 4, and 6 min.

The conditions were altered slightly to characterize the constructs with divarinic acid as the substrate. For M31, the time course was 0.5, 1, 1.5, and 2 min. For M23, the time course was 5, 10, 15, and 20 min, and for WT NphB the time course was 8, 16, 24, and 32 min. The enzyme concentration for the mutants was ~27 µM, and the concentration of WT NphB was ~35 µM.

### GC–MS characterization of isomer profile for WT NphB and M23

Samples were dissolved in 200 µL of ethyl acetate. Gas chromatography (GC)–MS measurements were carried out using an Agilent Model 7693 Autosampler, 7890B Gas Chromatograph, and 7250 Q-TOF Mass Selective Detector in the Electron Ionization mode. Sample injection was carried out in split mode with inlet temperature set to 280 °C. Separation was carried out on an Agilent HP5-MS column with dimensions 30 m × 250 × 0.25 µm. Ultra High Purity Grade He (Airgas) was used as carrier gas with the flow set to 1.1 mL/min in constant flow mode. The initial oven temperature was set to 120 ^o^C for 1 min followed by a 20 °C/min ramp to a final temperature of 300 ^o^C which was maintained for 4 min. A 3.0 min solvent delay was used. EI energy was set to 15 eV. The MSD was set to scan the 50–500 m/z range. Data collection and analysis were performed using Mass Hunter Acquisition and Qualitative Analysis software (Agilent).

Due to the increased temperature of the GC inlet, CBGA undergoes spontaneous decarboxylation as described by Radwan et al.^41^, resulting in an M+ ion at 316 m/z. The retention time corresponding to the 316 m/z ion for the CBGA standard was 10.48 min.

### Nonane-flow system for the extraction of CBGA from solution

A PyOx/PTA reaction was set up as detailed above. A 500 µL nonane overlay was added to the reaction in a 2 ml glass vial which was covered with 2 layers of breathable cell culture film. Two 18-gauge needles were inserted into a 15 mL falcon tube at the ~750 µL mark and the 3.5 mL mark. Luer locks to tubing connectors were connected to the needles and Viton tubing was connected to the other end of the luer lock. Eighteen-gauge needles were connected to the other end of the tubing via a luer lock connector and inserted through the mesh covering so they were only touching the nonane layer and not the reaction. In total, 2 mL of Tris buffer [pH 8.5] was added to the 15 mL conical tube, and 6 mL of nonane was added. The nonane was pumped through the system using a peristaltic pump (~1 mL/min) such that the nonane flowed from the top of the reaction, through the buffered solution (~18 cm tubing). The nonane pumped into the reservoir separated into the top layer of the 15 mL conical tube. The nonane from the top of the 15 mL conical tube was pumped into the top of the reaction vial (~55 cm tubing). This essentially diluted the CBGA throughout the system driving the diffusion of CBGA into the nonane layer and out of the reaction.

### Cloning CBDAS

A gene block of cannabidiolic acid synthase (CBDAS) with the signaling peptide was ordered from IDT codon optimized for *Pichia pastoris*. The signal sequence was removed by PCR amplifying from the 28th residue of the protein sequence (NPREN…) through the end of the protein, with overhangs compatible with the pPICZα vector. The PCR product was cloned into the pPICZα vector digested with EcoRI and XbaI using the Gibson cloning method. The product of the assembly reaction was transformed into BL21 Gold (DE3) cells, and a clone with the correct sequence was isolated. The plasmid was digested with PmeI for 2 h, and then purified using the Qiagen PCR purification protocol. The plasmid was transformed into Pichia pastoris X33 using electroporation. Immediately following electroporation, the cells were incubated in 1 mL of cold 1 M sorbitol and 1 mL of YPD media without shaking for 2 h. The cells were plated on YPDS plates with 500 µg/mL of zeocin. Colonies were screened using PCR for the presence of the CBDAS gene between the AOX1 promoter and terminator. For screening, the colonies were resuspended in 15 µL of sterile water and 5 µL of the resuspended colony was transferred into a PCR tube with 0.2% SDS. The samples were heated for 10 min at 99 °C, and then 1 µL was used as the template for PCR. Six colonies with positive colony PCR hits were screened for the expression of CBDAS.

### CBDAS-expression test

The six colonies were grown overnight at 30 °C in 25 mL of buffered complex glycerol medium (BMGY) to obtain a saturated culture. The overnight cultures were used to inoculate a 25 mL culture in BMGY media and grown to an OD of ~2. The cells were harvested by centrifugation at 2000 × *g* for 10 min. The cell pellet was resuspended in 90 mL of buffered minimal methanol yeast extract media, and incubated at 30 °C for 5 days. Each day, 1 mL of the culture was removed for SDS–PAGE analysis, and 500 µL of methanol was added to the remaining culture. On day 3 the cultures were screened for CBDAS activity. The 1 mL culture samples were centrifuged to pellet the cells (16,060 × *g*, 5 min). A 50 µL of the media was used in a subsequent activity assay, and the remainder of the media was stored at −80 °C in addition to the cell pellet. The assay conditions were as follows: 100 µL of 200 mM citrate buffer, 100 µM CBGA, 5 mM MgCl_2_, 5 mM KCl, 1 mM FAD, and 50 µL of the expression media in a final volume of 200 µL. The reactions were incubated overnight at room temperature and then extracted 3 times with 200 µL of ethyl acetate. The ethyl acetate extractions were pooled for each sample, and removed using a vacuum centrifuge. The samples were resuspended in 200 µL of methanol and analyzed by HPLC. All clones produced active CBDAS.

The culture from three clones (~300 mL total), was collected to obtain CBDAS activity. The cells were pelleted by centrifuging at ~3000 × *g* for 20 min at 4 °C. Then the supernatant was passed through a 0.22 µm filter. The media was concentrated and buffer exchanged into 100 mM citrate buffer pH 5.0 using a 50,000 molecular weight cut-off protein concentrator from Millipore.

### Production of CBDVA and CBDA

To convert the precursors CBGA and CBGVA into CBDA and CBGVA, respectively, a secondary reaction was set up with CBDAS. To produce CBDA, a PyOx/PTA enzymatic system was set up as detailed above to produce CBGA. After 24 h 200 µL of the nonane overlay from the CBGA reaction was transferred to a CBDAS reaction vessel. In the aqueous layer: 50 mM Hepes [pH 7.0], 5 mM MgCl_2_, 5 mM KCl, 25 µM FAD, 0.1 mg/mL CBDAS concentrate. The reaction was incubated at 30 °C with gentle shaking. Reactions were quenched at 12, 24, 48, 72, and 96 h.

To produce CBDVA, HPLC purified CBGVA was converted to CBDVA using CBDAS. The final reaction volume was 200 µL, with 50 mM Hepes [pH 7.0], 5 mM MgCl_2_, 5 mM KCl, 25 µM FAD and 0.1 mg/mL (total protein) of CBDAS concentrate. A 200 µL nonane overlay was added, and the reactions were incubated at 30 °C with gentle shaking. The reactions were quenched at ~24, 48, 72, and 96 h.

### Reporting summary

Further information on experimental design is available in the Nature Research Reporting Summary linked to this article.

## Supplementary information

Supplementary Information

Peer Review File

Reporting Summary

Supplementary Data 1

Source Data

Description of Additional Supplementary Files

## Data Availability

All data generated or analyzed in this study are in this published article and its Supplementary materials. The authors will make every reasonable effort to provide data and materials described herein upon request. A reporting summery for this article is available as a Supplementary Information file. The source data underlying Figs. 2A–D, 3B, C, 4A, C, Supplementary Figures 2 and 4 and Supplementary Table 5 are provided as a Source Data file.
