# Author Correction: A cell-free platform for the prenylation of natural products and application to cannabinoid production

**DOI:** 10.1038/s41467-019-10436-1

**Published:** 2019-05-24

**Authors:** Meaghan A. Valliere, Tyler P. Korman, Nicholas B. Woodall, Gregory A. Khitrov, Robert E. Taylor, David Baker, James U. Bowie

**Affiliations:** 1 Department of Chemistry and Biochemistry, Molecular Biology Institute, UCLA-DOE Institute, University of California, Los Angeles, 90095 CA USA; 2 Department of Biochemistry, Institute for Protein Design, University of Washington, Seattle, 98105 WA USA

**Keywords:** Biocatalysis, Enzymes, Metabolic engineering, Biocatalysis

Correction to: *Nature Communications* 10.1038/s41467-019-08448-y, published online 4 Feb 2019.

In the original version of this Article, the genotype of the M30 mutant presented in Fig. 3b was given incorrectly as Y288V/A232S, and the M31 mutant was given incorrectly as M1/A232S. The correct genotype of the M30 mutant is Y288A/A232S and for M31 it is Y288V/A232S. In addition, to keep consistency in genotype formatting, the genotype of the M27 mutant should be Y288V/G286S. The errors have been corrected in both the PDF and HTML versions of the Article.

