# Supplementary material for: A cell-free platform for the prenylation of natural products and application to cannabinoid production
Source: Nat Commun. 2019 Feb 4;10:565. doi: 10.1038/s41467-019-08448-y (PMC6362252; doi:10.1038/s41467-019-08448-y)
Supplement: Supplementary file 1 — Supplementary Information [file 41467_2019_8448_MOESM1_ESM.pdf]

A cell-free biosynthetic platform for the prenylation of natural products with applications to cannabinoid production

Valliere *et al.*

Supplementary Table 1: Enzymes used in the enzymatic platform.

	Enzyme Abb.	Full Name	Organism	Amount Added to Rxn (mg/mL)	Acquisition Number
**1**	Hex	Hexokinase	*S. cerevisiae*	0.02	Sigma Aldrich
**2**	Pgi	Glucose-6-phosphate isomerase	*G. thermodenitrificans*	0.48	ABO6822
**3**	PfkA	Phosphofructokinase	*G. stearothermophilus*	0.18	KOR92562
**4**	Fba	Fructose-1,6-bisphosphate aldolase	*S. aureus*	0.03	BAR10119
**5**	TpiA	Triose phosphate isomerase	*G. stearothermophilus*	0.16	KOR95273
**6**	Gap	Gald-3-P dehydrogenase	*E. coli K12*	0.07	NP_416293
**7**	mGap	Gald-3-P dehydrogenase D34A/L35R/T36K	*G. stearothermophilus*	0.18	NP_416293
**8**	NoxE	NADH Oxidase	*L. lactis*	0.25	WP_015425842
**9**	Pgk	Phosphoglycerate Kinase	*G. stearothermophilus*	0.06	NP_415276
**10**	dPgm	Phosphoglycerate Mutase (2,3 BPG dependent)	*E. coli K12*	0.29	NP_417259
**11**	Eno	Enolase	*E. coli K12*	0.08	KOR95272
**12**	PykF	Pyruvate Kinase (FBP dependent)	*E. coli K12*	0.37	NP_416191
	PDH	Pyruvate Dehydrogenase	*E. coli K12*	0.99	
		AceE			NP_414656
		AceF			NP_414657
		Lpd			NP_414658
**13**	PyOx	Pyruvate Oxidase	*A. viridans*	1 U	AG Scientific
**14**	PTA	Acetyl-phosphate transferase	*G. stearothermophilus*	0.06	WP_053532564
**15**	PhaA	Acetyl-CoA acetyltransferase	*R. eutropha*	0.12	CAJ92573
**16**	HMGS A110G	HMG-CoA Synthase A110G	*E. faecalis*	0.18	WP_010785222
**17**	HMGR	HMG-CoA Reductase	*E. faecalis*	0.16	AAG02439
**18**	MVK	Mevalonate Kinase	*M. mazei*	0.14	AAM31458
**19**	PMVK	Phosphomevalonate Kinase	*S. pneumonia*	0.2	WP_000562411
**20**	MDC	Diphosphomevalonate Kinase	*S. pneumonia*	0.19	NP_357933
**21**	IDI	Isopentyl-PP Isomerase	*E. coli K12*	0.3	NP_417365
**22**	FPPS S82F	Farnesyl-PP synthase S82F	*G. stearothermophilus*	0.09	KOR95521
**23**	NphB	Aromatic prenyltransferase	*Streptomyces sp. CL190*	Variable	BAE00106.1
**24B**	CBDAS	Cannabidiolic Acid Synthase	*C. sativa*		AKC34419
**25**	Ppase	Pyrophosphatase	*G. stearothermophilus*	0.11	O05724
**26**	Cat	Catalase	*C. glutamicum*	0.1 U	Sigma Aldrich
	GorA	Glutathione Reductase	*E. coli K12*	0.06	NP_417957

The cells highlighted in yellow indicate proteins that were only added to the PDH reactions and not to the PyOx/PTA reactions

Supplementary Table 2: Rosetta suggested mutants varying initial olivetolate position.

	Olivetolate P1					Consensus Mutations	Olivetolate P2					Consensus Mutations	Olivetolate P3					Consensus Mutations
Prediction #	1	2	3	4	5	Group A	6	7	8	9	10	Group B	11	12	13	14	15	Group C
V49	I	T	I	I	I	**I**	T	S	S	S	T	**S**	N		N	N	S	**N**
M162													C	C	C	C	C	**C**
F213	N	N	N	N	N	**N**	N	N	N	N	N	**N**	N	N	N	N	N	**N**
A232	N	N	N	N	S	**N**	S	S	S	S	S	**S**						
I234	T	T	T	T	T	**T**	T	T	T	T	T	**T**	T	T	T	T	T	**T**
V271	N	H	N	N	N	**N**	N	N	N	N	N	**N**	N	N	N	N	N	**N**
G286								S	S	S		**S**	S		S	S	S	**S**
Y288	A	D	A	A	H	**A**	N	N	N	N	N	**N**	S	A	S	S	N	**S**
L298	I	I	I	I	I	**I**	R	R	R	R	R	**R**	R	R	R	R	R	**R**
**Energy Score**						**-404**						**-410**						**-405**

	Olivetolate P4					Consensus Mutations	Olivetolate P5					Consensus Mutations	Olivetolate P6					Consensus Mutations
Prediction #	16	17	18	19	20	Group D	21	22	23	24	25	Group E	26	27	28	29	30	Group F
V49	T	N	T	N	T	**T**	S	S	I	S	S	**S**	G	G	G	S	S	**G**
M162							R	R	R	R	R	**R**	R	R	R	R	R	**R**
F213	G	G	G	G	G	**G**	N	N	N	N	N	**N**	N	N	N	N	N	**N**
A232							N	S	N	S	N	**S**	S	S	S	S	S	**S**
I234	T	T	T	T	T	**T**	T	T	T	T	T	**T**	T	T	T	T	T	**T**
V271	H	N	H	N	H	**H**	N	N	S	N	N	**N**	A	N	N	A	A	**N**
G286																		
Y288	D	S	N	S	D	**N**	N	N	N	N	N	**N**	N	N	N	A	A	**N**
L298	I	I	I	I	I	**I**	I	A	N	A	N	**A**	G	V	V	G	G	**V**
**Energy Score**						**-402**						**-403**						**-398**

Olivetolate (OA) was placed into the active site at NphB based off the location of 1,6 DHN with the C3 carbon 3.7 angstroms above the C1 carbon of GPP. We tested 6 different OA starting positions, and ran each scenario 5 times. The resulting NphB mutants are shown in the table above.

Supplementary Table 3: Evaluation of mutation significance according to the Rosetta energy score function.

Olivetolate Position 1

Amino Acid Position	Consensus Mutations Group A	WT	Energy Score of Mutant --> WT	Energy Difference
49	I	V	-403	1
213	N	F	-391	13
232	N	A	-401	3
234	T	I	-382	22
271	N	V	-395	9
288	A	Y	-392	12
298	I	L	-404	0

Mutations with largest effect

I234T
F213N
Y288A

Olivetolate Position 2

Amino Acid Position	Consensus Mutations Group B	WT	Energy Score of Mutant --> WT	Energy Difference
49	S	V	-394	16
213	N	F	-402	8
232	S	A	-409	1
234	T	I	-404	6
271	N	V	-397	13
286	S	G	-409	1
288	N	Y	-401	9
298	R	L	-408	2

Mutations with largest effect

V49S
V271N
Y288N

Olivetolate Position 3

Amino Acid Position	Consensus Mutations Group C	WT	Energy Score of Mutant --> WT	Energy Difference
49	N	V	-391	14
162	C	M	-404	1
213	N	F	-390	15
234	T	I	-400	5
271	N	V	-396	9
286	S	G	-404	1
288	S	Y	-394	11
298	R	L	-403	2

Mutations with largest effect

F213N
V49N
Y288S

Olivetolate Position 4

Amino Acid Position	Consensus Mutations Group D	WT	Energy Score of Mutant --> WT	Energy Difference
49	T	V	-401	1
213	G	F	-98	304
234	T	I	-372	30
271	H	V	-398	4
288	N	Y	-381	21
298	I	L	-401	1

Mutations with largest effect

F213G
I234T
Y288N

Olivetolate Position 5

Amino Acid Position	Consensus Mutations Group E	WT	Energy Score of Mutant --> WT	Energy Difference
49	S	V	-398	5
162	R	M	-402	1
213	N	F	-318	85
232	S	A	-327	76
234	T	I	-398	5
271	N	V	-391	12
288	N	Y	-390	13
298	A	L	-394	9

Mutations with largest effect

F213N
A232S
Y288N

Olivetolate Position 6

Amino Acid Position	Consensus Mutations Group F	WT	Energy Score of Mutant --> WT	Energy Difference
49	G	V	-383	15
162	R	M	-398	0
213	N	F	-388	10
232	S	A	-388	10
234	T	I	-388	10
271	N	V	-390	8
288	N	Y	-367	31
298	V	L	-397	1

Mutations with largest effect

Y288N
V49G

For each group of consensus mutants from Supplementary Table 3, we returned one mutation back to WT, and the remainder were not changed. We used Rosetta to calculate the difference in the energy score when the residue was set back to WT. We repeated that for each Rosetta mutation, and the results are shown in the table above. The mutations contributing to the largest change in energy are highlighted in yellow.

Supplementary Table 4: NphB mutants.

Construct Name	Mutations
**M1**	Y288A
**M2**	Y288N
**M3**	Y288A, F213H
**M4**	Y288A, F213N
**M5**	Y288N, V49S
**M6**	Y288S, V49N
**M7**	Y288A, V49S
**M8**	Y288N, V49T
**M9**	Y288N, I234T
**M10**	Y288N, G286S
**M11**	Y288N, F213N, V49G
**M12**	Y288A, F213N, I234T
**M13**	Y288S, F213N, V49N
**M14**	Y288N, F213G, I234T
**M15**	Y288A, F213N, A232S
**M16**	Y288N, F213N, A232S
**M17**	Y288N, F213G, V49T
**M18**	Y288N, V49S, V271N
**M19**	Y288N, F213N, V49S, V271N
**M20**	Y288N, F213G, V49T, V271H
**M21**	Y288N, F213N, V49S, I234T, A232S, V271N
**M22**	Y288N, F213G, V49T, I234T, V271H, L298I
**M23***	Y288A, G286S
**M24***	Y288A, G286S, A232S
**M25***	Y288A, G286S, A232S, F213H
**M27***	Y288V, G286S
**M28***	Y288V, G286S, A232S
**M30***	Y288A, A232S
**M31***	Y288V, A232S

The NphB construct name, and the amino acid mutations are shown above. The asterisk (*) denotes constructs that were added after the first round was screened.

Supplementary Table 5: Kinetic parameters for NphB mutants.

**Construct**	**k_{cat} (min^{-1})**	**K_M (mM)**	**k_{cat}/K_M (min^{-1} mM^{-1})**
WT NphB	0.0021 ± 0.00008<br>0.0047 ± 0.0003 [b]	0.64 ± 0.08<br>0.88 ± 0.2 [b]	0.0033 ± 0.0005<br>0.005 ± 0.001 [b]
NphB M1	0.061 ± 0.003	0.58 ± 0.11	0.11 ± 0.02
NphB M10	0.79 ± 0.02	0.34 ± 0.02	2.4 ± 0.2
NphB M23	1.58 ± 0.05<br>0.48 ± 0.07 [b]	0.45 ± 0.05<br>2.4 ± 0.6 [b]	3.5 ± 0.4<br>0.2 ± 0.06 [b]
NphB M30	1.05 ± 0.05	0.200 ± 0.04	4.2 ± 0.9
NphB M31	1.30 ± 0.05<br>6.0 ± 0.8 [b]	0.12 ± 0.02*<br>1.8 ± 0.5 [b]	10.8 ± 2.1<br>3.3 ± 1 [b]

Each construct was evaluated for its CBGA production with olivetolate (OA) as a substrate. WT, M23 and M31 were evaluated with divarinic acid as well. The symbol ([b]) indicates that divarinic acid was the substrate. Source data are provided as a Source Data file.

Supplementary Table 6: Primers used in this study

Primer direction	Primer sequence (5'->3')
For amplification of CBDAS without signal peptide	
Forward	ctcgagaagagagaggctgaagcatcgatgaatcccagagagaactttcttaagtgtttc
Reverse	atcctcttctgagatgagtttttgttctagggaatgcctgtgcctaggtagagggggtat
For amplification of Gs PTA	
Forward	ggtgccgcgcggcagccatatgacaaccgatttatttacggcattaaaagc
Reverse	cgcagtcgcttggggagtaactcgagcaccaccaccaccaccactg

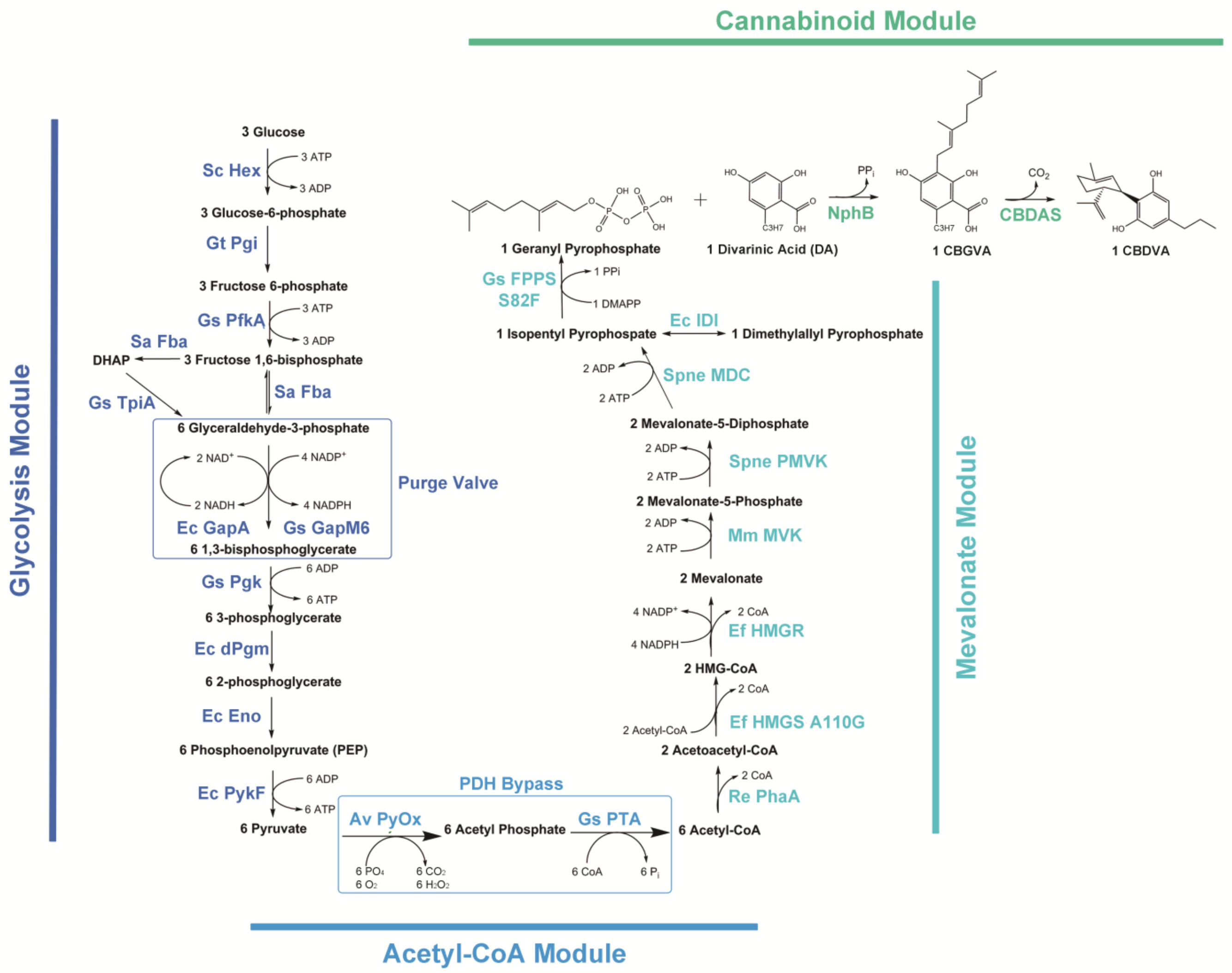

Supplementary Figure 1: Full reaction schematic for the enzymatic synthesis of cannabinoids. Glucose is broken down to pyruvate through a modified glycolysis pathway (dark blue) that includes a purge valve system. The purge valve (boxed) allows carbon flux to continue through the glycolysis pathway without building up excess NADPH. Pyruvate is converted to acetyl-CoA through the PDH bypass outlined in light blue. Acetyl-CoA is then converted into GPP via the mevalonate pathway (aqua). Finally, the GPP from the mevalonate pathway is used to prenylate aromatic polyketide. Shown here is the prenylation of olivetolate to produce CBGA; however, olivetolate could be replaced with a wide range of aromatic substrates to generate various prenylated products. A prenylated product like CBGA can be converted into a variety of cannabinoids. An example of the conversion of CBGA into CBD by the action of CBDAS and a spontaneous decarboxylation is shown.

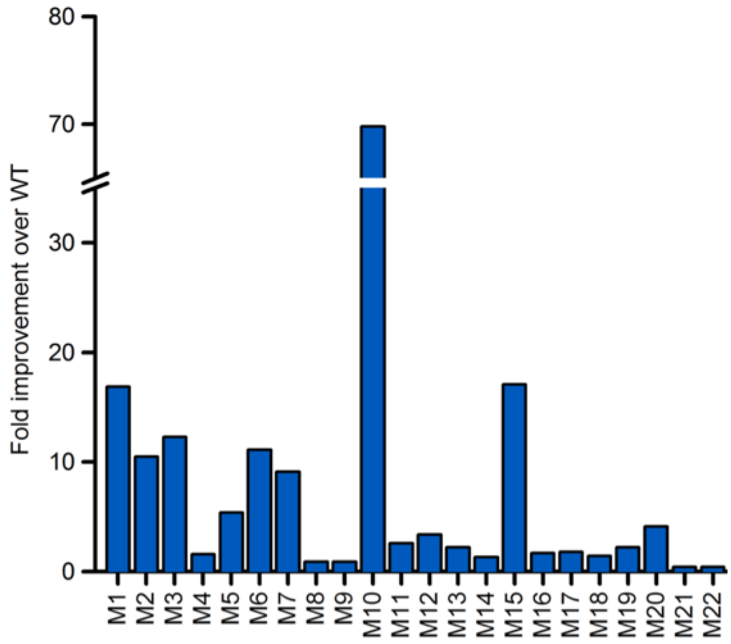

Supplementary Figure 2: Activity screen of NphB constructs. NphB constructs from the initial round were expressed and purified and assayed for CBGA production. The constructs are shown on the x-axis, and their activity relative to WT activity is shown on the y-axis. Source data are provided as a Source Data file.

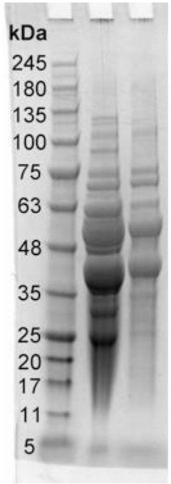

Supplementary Figure 3: SDS-PAGE analysis of CBGA cell-free system precipitate. Lane 1: 1 kb DNA ladder from BioPioneer. Lane 2: Enzyme mastermix before it was added to the reaction. Lane 3: The washed precipitate from the reaction at 24 hours.

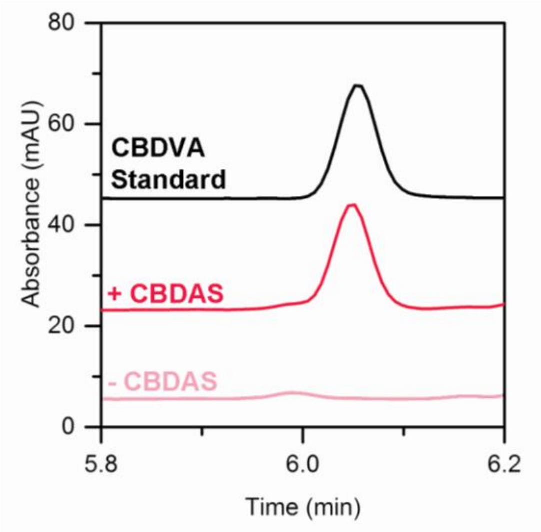

Supplementary Figure 4: Cannabidiolic acid synthase (CBDAS) converts CBGVA into CBDVA. HPLC chromatogram of reaction extracts of CBGVA in the presence (red) and absence (pink) of CBDAS compared to a CBDVA standard (black). Source data are provided as a Source Data file.

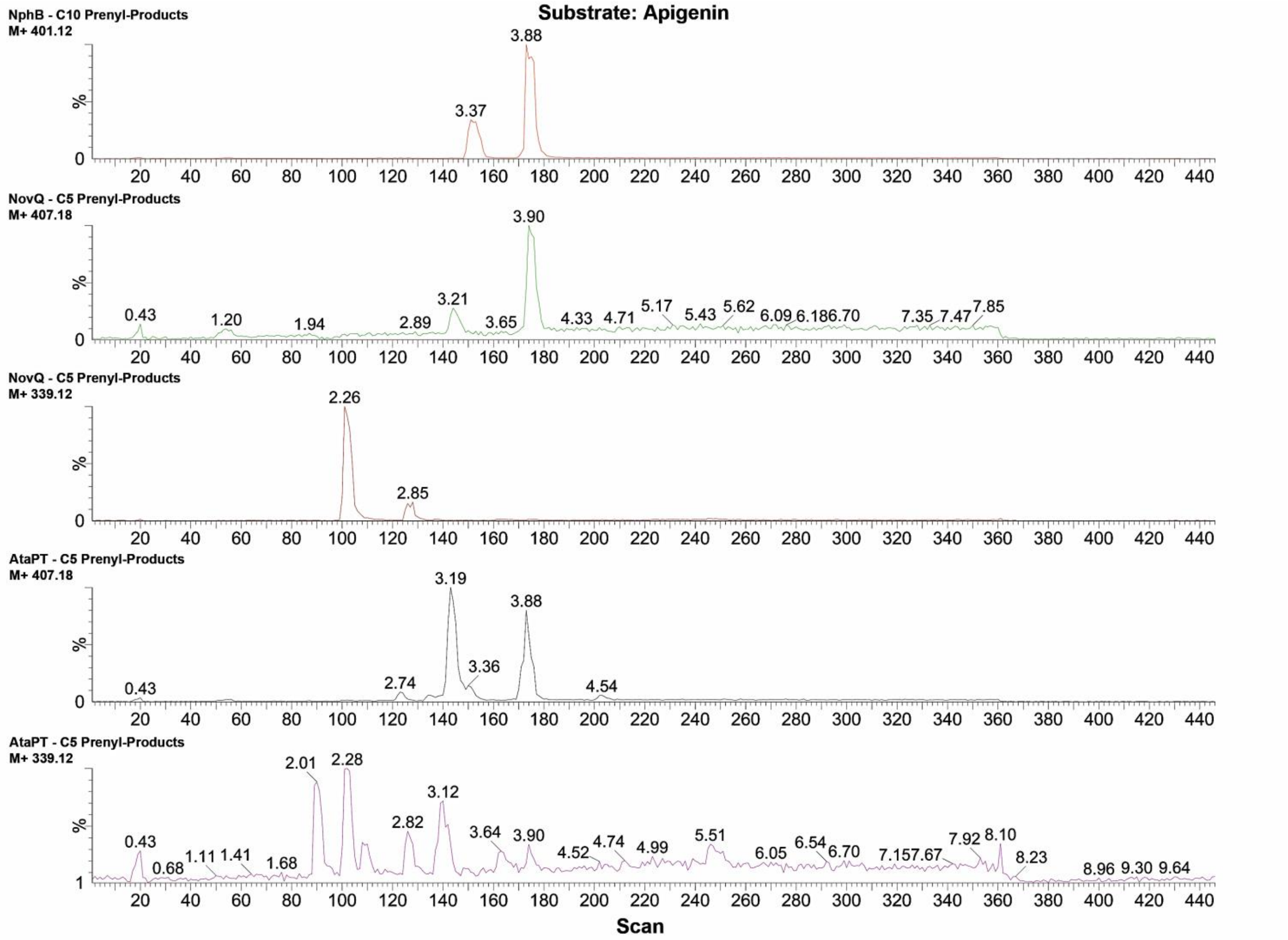

Supplementary Figure 5: LC-MS chromatogram with extracted for masses corresponding to prenyl-apigenin products. Each panel is labeled for the enzyme that produced the prenyl-products, and the m/z that corresponds to the prenyl-products. AtaPT and NovQ demonstrate both mono and di-prenylation.

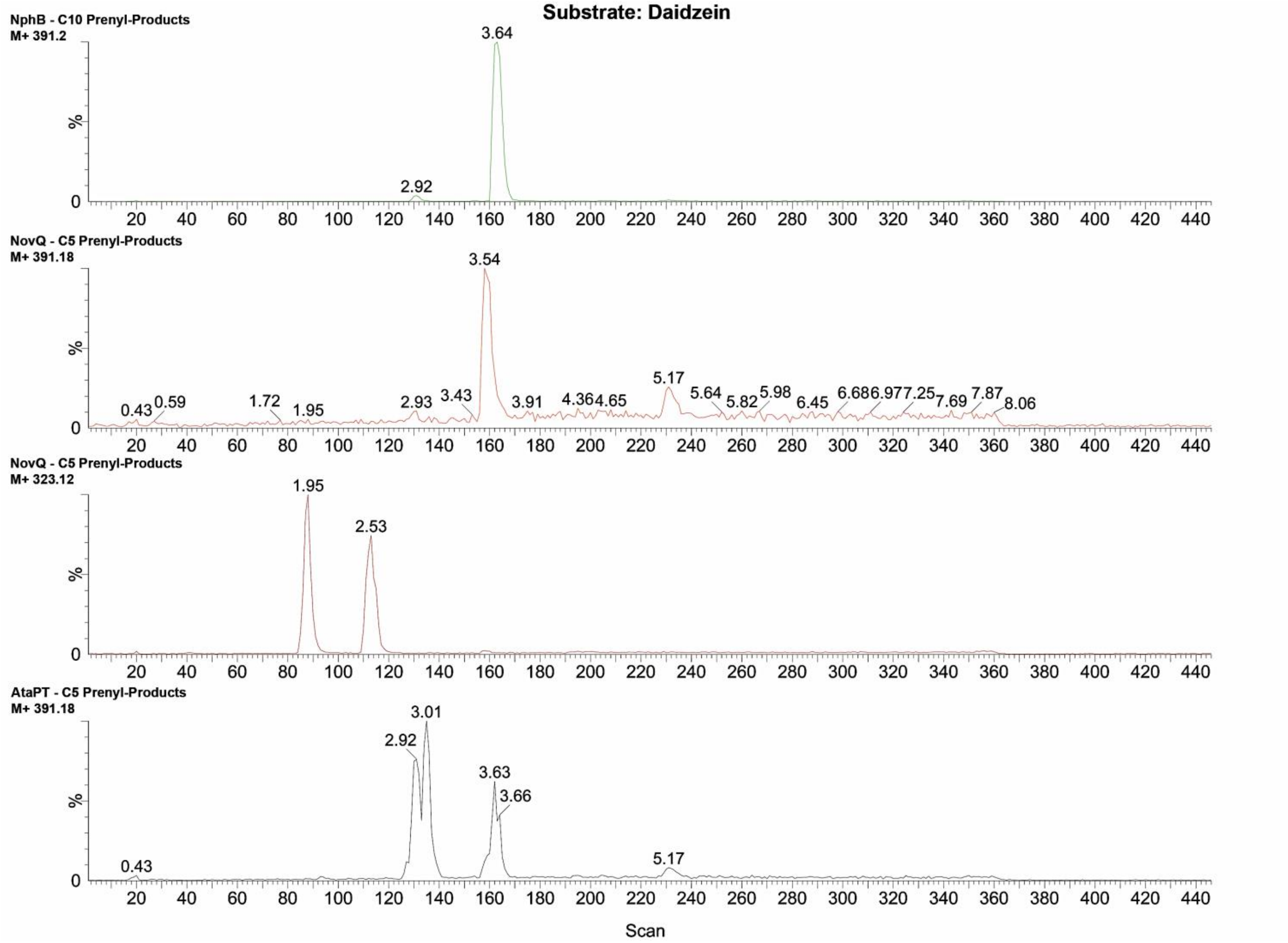

Supplementary Figure 6: LC-MS chromatogram with extracted for masses corresponding to prenyl-daidzein products. Each panel is labeled for the enzyme that produced the prenyl-products, and the m/z that corresponds to the prenyl-products. NovQ demonstrate both mono and di-prenylation, whereas AtaPT demonstrates only diprenylation.

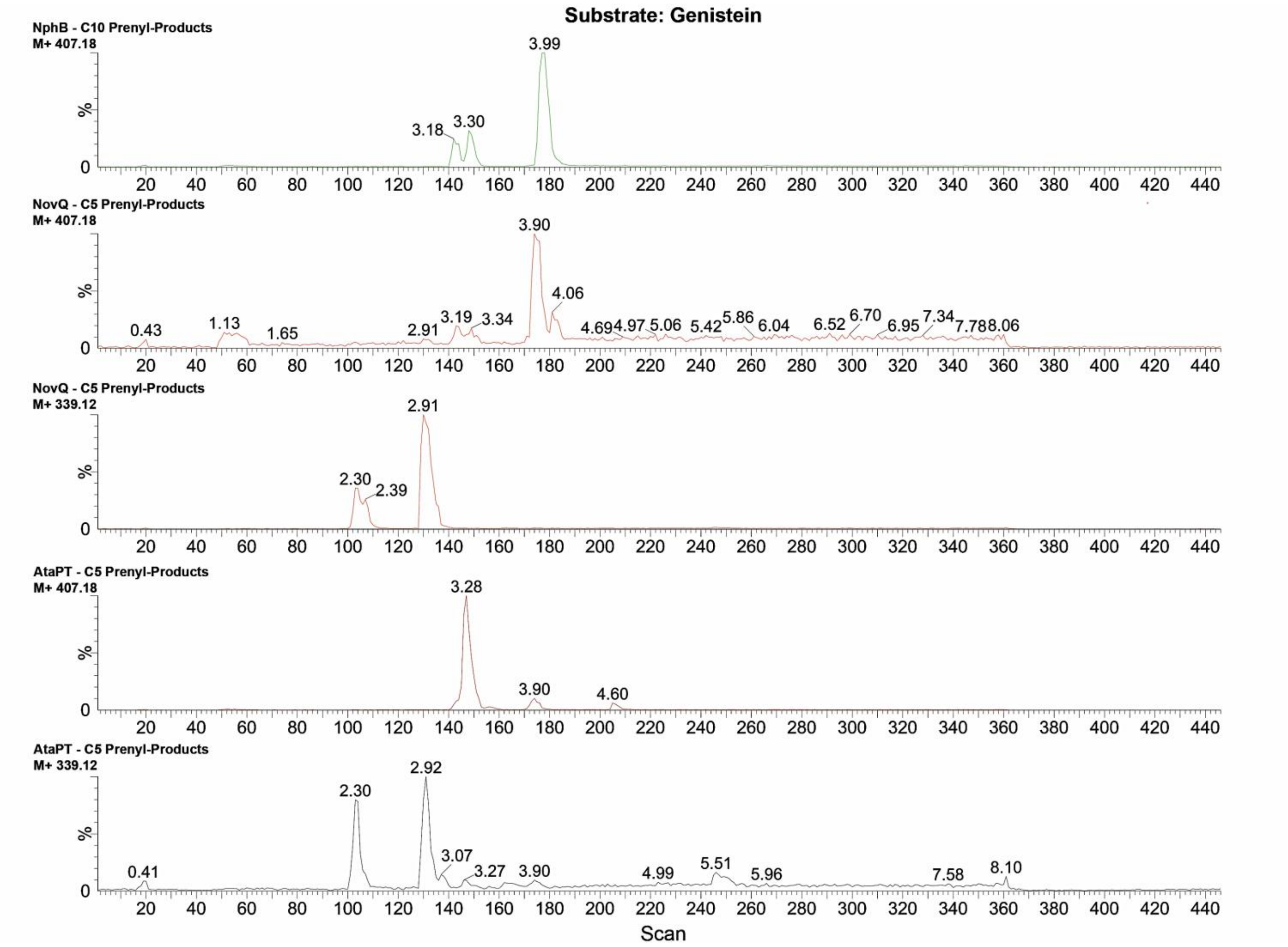

Supplementary Figure 7: LC-MS chromatogram with extracted for masses corresponding to prenyl-genistein products. Each panel is labeled for the enzyme that produced the prenyl-products, and the m/z that corresponds to the prenyl-products. AtaPT and NovQ demonstrate both mono and di-prenylation.

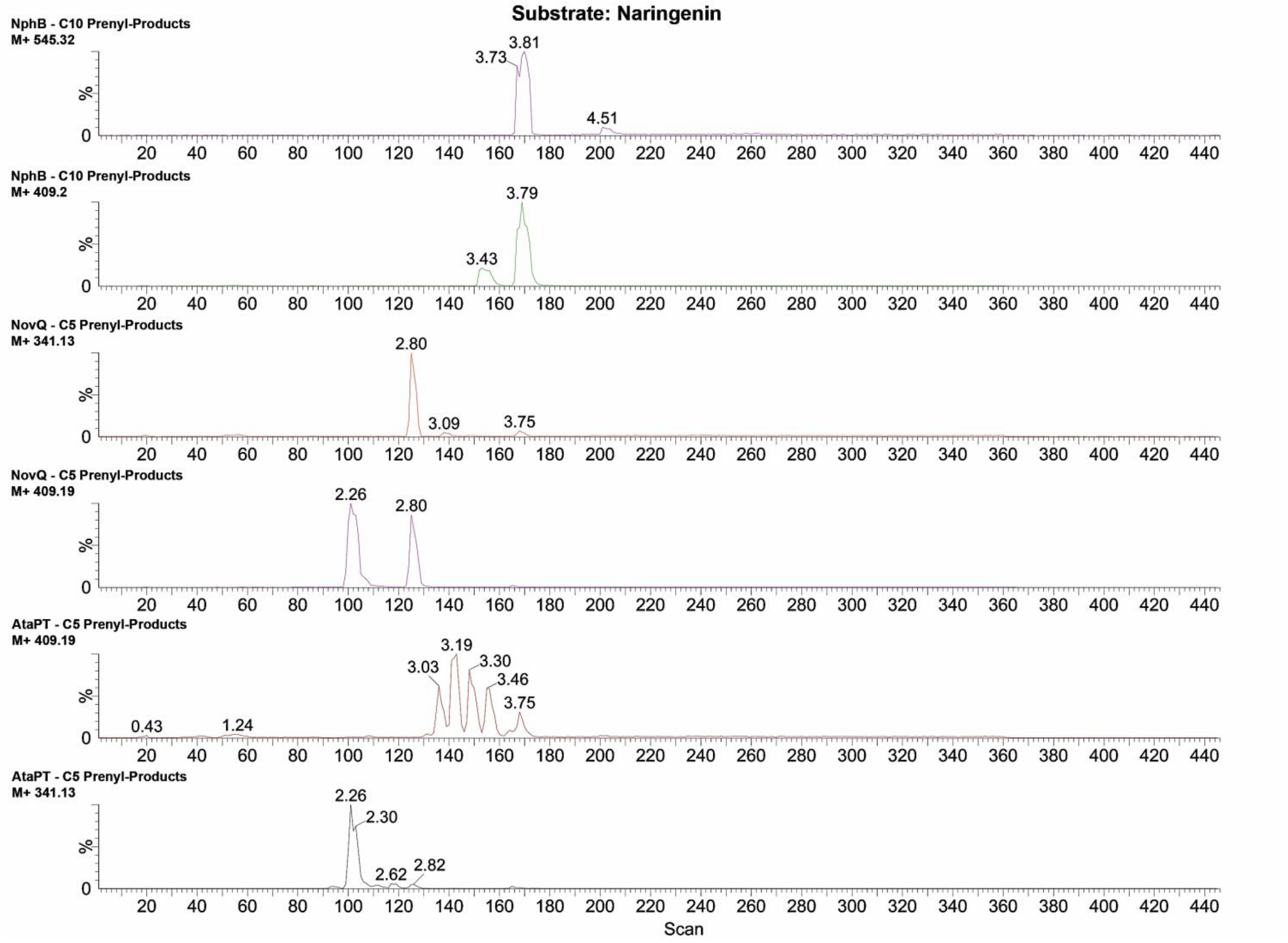

Supplementary Figure 8: LC-MS chromatogram with extracted for masses corresponding to prenyl-naringenin products. Each panel is labeled for the enzyme that produced the prenyl-products, and the m/z that corresponds to the prenyl-products. AtaPT and NovQ demonstrate both mono and di-prenylation, NphB demonstrated both mono and di-geranylation.

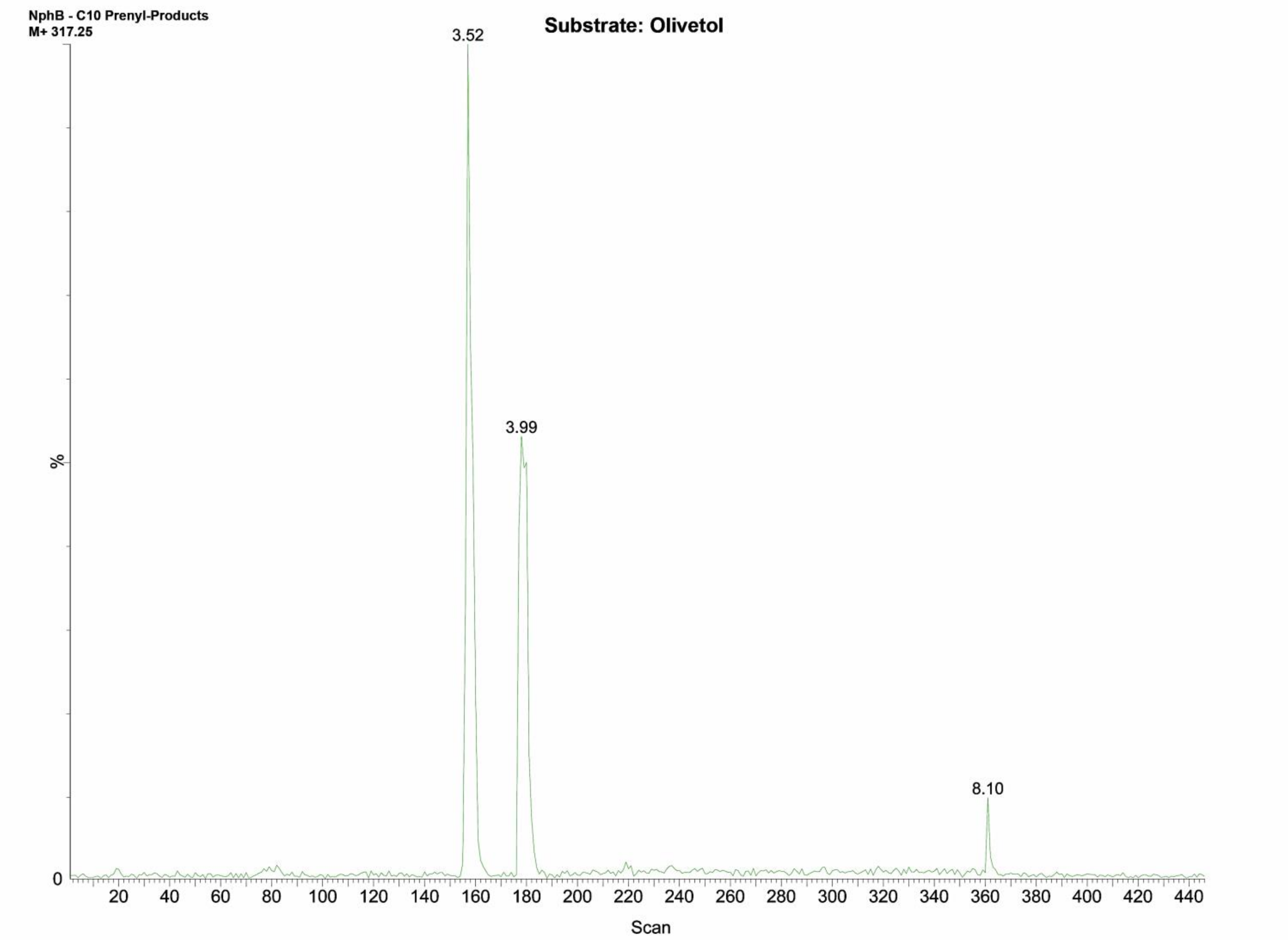

Supplementary Figure 9: LC-MS chromatogram with extracted for masses corresponding to prenyl-olivetol products. This panel is labeled with the enzyme that produced the prenyl-products, and the m/z that corresponds to the prenyl-products.

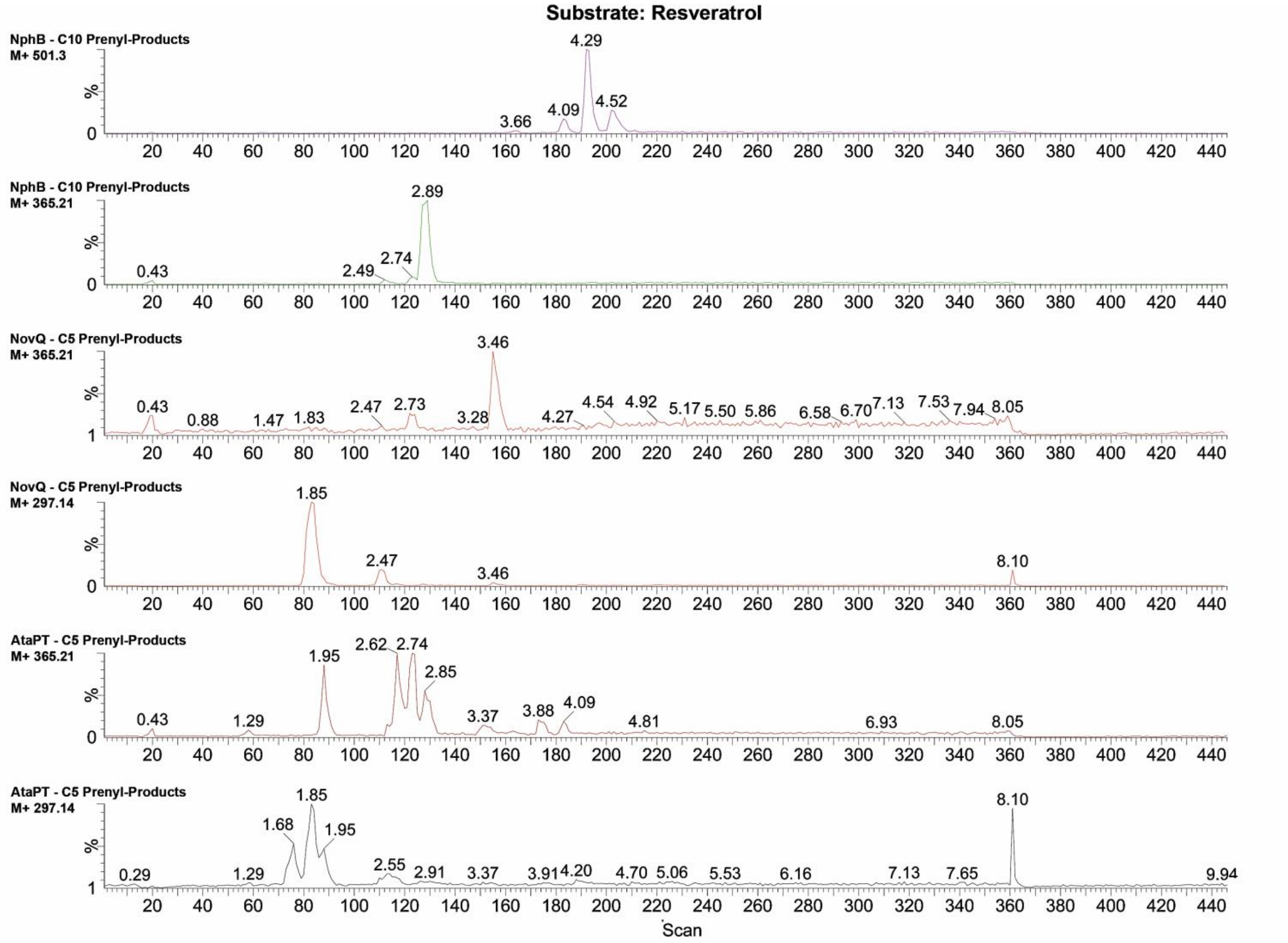

Supplementary Figure 10: LC-MS chromatogram with extracted for masses corresponding to prenyl-resveratrol products. Each panel is labeled for the enzyme that produced the prenyl-products, and the m/z that corresponds to the prenyl-products. AtaPT and NovQ demonstrate both mono and di-prenylation, and NphB demonstrates both mono and di-geranylation.

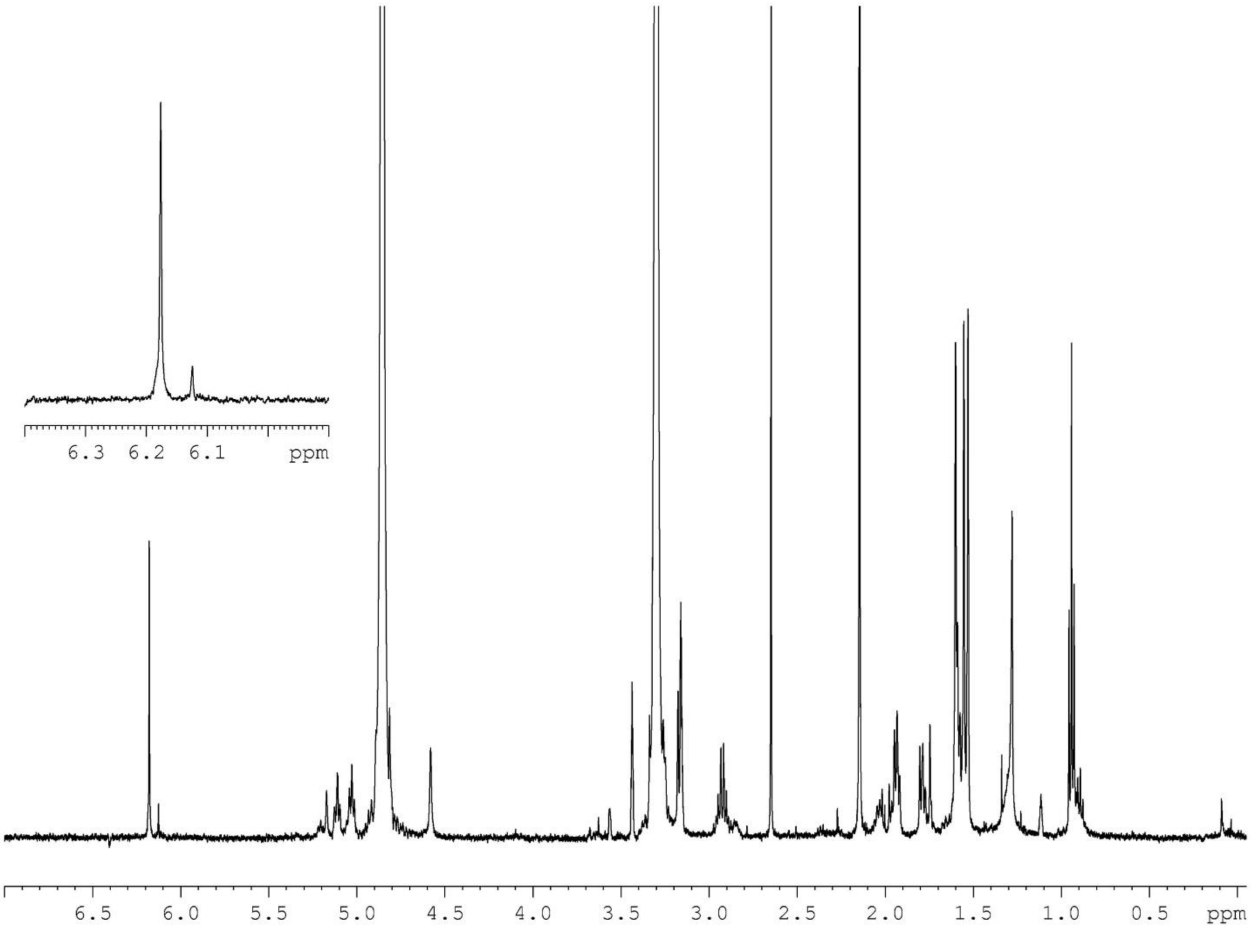

Supplementary Figure 11: Proton NMR Spectrum of CBGVA in CD_3OD. The entire spectra is displayed, and the inlet zooms in on the aromatic proton at ~6.2 ppm.
