# Supplementary material for: A cell-free platform for the prenylation of natural products and application to cannabinoid production
Source: Nat Commun. 2019 Feb 4;10:565. doi: 10.1038/s41467-019-08448-y (PMC6362252; doi:10.1038/s41467-019-08448-y)
Supplement: Supplementary file 6 — Description of Additional Supplementary Files [file 41467_2019_8448_MOESM6_ESM.pdf]

Description of Additional Supplementary Files

File Name: Supplementary Data 1

Description: Gene blocks used in this work
