# Supplementary material for: A cell-free platform for the prenylation of natural products and application to cannabinoid production
Source: Nat Commun. 2019 Feb 4;10:565. doi: 10.1038/s41467-019-08448-y (PMC6362252; doi:10.1038/s41467-019-08448-y)
Supplement: Supplementary file 2 — Peer Review File [file 41467_2019_8448_MOESM2_ESM.pdf]

Journal peer review information: *Nature Communications* thanks Olive Kayser and the other anonymous reviewer(s) for their contribution to the peer review of this work. [Peer reviewer reports are available.]

Reviewers' comments:

Reviewer #1 (Remarks to the Author):

The paper is well written and highlights an important aspect of aromatic C prenylation by NphB. The title is in some way misleading due to the fact that most of the work presentated is about prenylation done by an engineered NphB and less about cell free bioengineering.

My main concenr is about the not sufficient analytics. Basic questions is not solved: Is the detected CBGA and CBVA correlated with the proposed structure? Zirpel et al, (2018) show also various isomers and O-prenylation. It is very likely that the presented isoforms are O-prenylated products. Even by C-prenylation two mono prenylation sites are there. By MS we can exclude di-prenylation, but even that is not fully excluded. These questions can only be fully answered by NMR. Due to the high titers claimed, I suggest isolation and H/C NMR based structure elucidation before publication.

The proposed cell-free system is not innovative and has been used several times in ordinary labs determining enzyme kinetics. I am surprised to read here about an innovative system for industry, what is not true. The experimental design is a simple biotransformation that is by the way not fully characterized by bioengineers standards. Here a full process design is needed. Without that, I can not see what is outstanding to be published in Nature Com.

The effect of a 1000 fold increased the activity of NphB is impressive. Mentioned facts like non-membrane binding and cytosolic activity are known. In the end, it is a protein engineering strategy in a rational way that is also not outstanding and a simple straightforward approach.

To sum up: Data provided are of high interest but are not outstanding to be published in Nature Communication. Cell-free production is no real option in the industry due to the fact that feeding all relevant precursors (GPP, OA etc) is too costly. There is no superior information about the question of energy supply and smart engineered substrate supply. Is it not clear where the focus is, is it about NphB optimization or cell free production.

Reviewer #2 (Remarks to the Author):

In this manuscript the authors present a cell-free system for the production of the prenylated cannabinoid precursors, CBGA and CBGVA. They show that other molecules can be prenylated with the cell-free system, but to a lesser extent. In addition, the authors use computational modeling of protein-substrate interactions to identify enzyme mutations to improve kinetics in the cell-free reactions. The combination of protein design and synthetic biochemistry is timely, and the science addresses an important limitation in the synthesis of prenylated molecules. However, there are several areas where the manuscript falls short, which lessened my enthusiasm. One issue, for example, is that the title and abstract made me expect/want that the authors had made cannabinoids, but they did not (they made cannabinoid acids which would need to be decarboxylated to make cannabinoids). This paper was a step along the path to those molecules, but is really about prenylation. While I do not believe the manuscript in its current form is ready, if the authors address the concerns below, this manuscript could rise to the level needed to be accepted in a journal like Nature Communications.

Major Concerns:

- The authors argue that they have a cell-free system that makes cannabinoids, yet cannabinoids are not exactly made in the study. The authors make one cannabinoid acid, CBDVA, which is given only one sentence in the main text and the unquantified data is placed in the supplement. This was disappointing and made it feel like the authors oversell the data. The manuscript could be improved by

carefully editing the text to accurately reflect the data presented.

- While this would require additional experimentation, I believe that the authors should make "therapeutically relevant cannabinoids" or their acids (THCA, CBDA, etc.) to warrant publication in this journal.

- Line 91, the authors state that Enzyme assays revealed that PDH is inhibited by 1,6 DHN and other molecules. What about the panel of molecules used in Fig 2C? The authors should show PDH activity for all these molecules. In addition, Fig 2A seems to suggest that PDH activity might be improved by the addition of OA, their molecule of interest. This would suggest that they shouldn't switch to the bypass system. Can the authors please clarify and did they try the final syntheses without the bypass? What are the results?

- Is the PDH bypass different than the author's previous work? Why wouldn't the authors start with the bypass system rather than showing us the PDH is less efficient? This doesn't seem like a new finding and seem supplemental to the stated goals of their manuscript.

- Figure 2 is not properly put together. The legend doesn't correspond with the figures shown. For instance , I believe that 2A is described as 2B in the legend and vice versa. In addition, throughout the text these figures and their panels are not properly referenced making it difficult to read the manuscript. For instance, line 92 should be 2A not 2B; line 98 should be 2D not 2C. Careful editing needs to be carried out.

- Figure 2C tests aromatic substrates with 3 different prenyl transferases in the bypass system. The authors should test these substrates and transferases under the PDH system as panel 2A suggests that for some of these aromatics it might work better. In addition, the asterisks denote not determined in the figure. Just to be clear this means the chromatography had no distinguishable peak or these weren't tested?

- Please be clearer in the legend that Figure 2D shows two different reaction setups in that different substrates are added. Presenting this in one graph makes it look like in one reaction you made prenylated DHN and CBGA.

- Should ROSETTA be in all caps?

- Line 112, a side-product is mentioned but the specifics should be stated. Did you observe side-products in the data? Can the data be provided?

- Lines 117-126 describes several conclusions drawn from data that is not referenced. I believe I was supposed to look at Figure S2. This should be a main body figure as the engineering of this enzyme is one of the more significant findings from this study.

- Line 132, the natural prenyltransferase is mentioned. What is its activity?

- Figure 4A should show WT NphB - CBGVA as well in order to compare. Also I believe this should be referenced on line 138.

- Figure 4B is a schematic and the measured data should be shown alongside it. It is mentioned that 1.25 g/L of CBGA was made, but a time course similar to 4A should be shown. In addition, the whole point of this more complicated reaction setup was to alleviate precipitation of enzymes. Are there no longer preciptates in the reactions? How long do the reactions now last? This data should be provided.

- Several of the supplementary figures (i.e. Figures S5-S10) are not, but should be, referenced in the text.

- The authors should cite their previous work on line 74.

- The authors should include exact conditions of their reactions or point to the methods section. Line 88 just says excess glucose.

Reviewer #3 (Remarks to the Author):

This article aims to construct cell-free enzymatic prenylating system for better production of important medicinal compounds. The cell-free system could break through the obstacles caused by membrane barriers, substance toxicities, etc. The approach studied in this article could provide a robust platform for producing chemicals that are hard to be made in living cells. This study is very interesting and also useful, but as currently presented, this manuscript needs to be further improved.
1. Glycolysis module, Acetyl-CoA module, and mevalonate module have been studied by many other researchers. Are there any innovative or obviously different research contents in this study?
2. Are there any other possible methods for PDH bypass, besides two enzymes PyOx and PTA?
3. The highlights of this article look like the construction of PDH bypass and the engineering of NphB. The depth of this cell-free platform needs to be increased, such as addressing the key challenges in this research field. More insightful information needs to be presented.
4. How about the solubility of all expressed enzymes in this study?
5. Have authors tried basic reaction parameters such as different temperatures and different pH values?
6. Cofactors and energy substrates have significant impacts on pathway activities. Have authors done any theoretical calculation or experiments for better control of cofactor and energy levels?
7. Line 156: Two periods.
8. Results in this study should be compared with previous studies by other researchers. Please indicate how the results relate to expectations and to earlier research.
9. More application examples need to be provided to prove the effectiveness of this cell-free prenylation platform.

Response to Reviewer comments

Reviewer #1 (Remarks to the Author):

The paper is well written and highlights an important aspect of aromatic C prenylation by NphB. The title is in some way misleading due to the fact that most of the work presentated is about prenylation done by an engineered NphB and less about cell free bioengineering.

We are somewhat taken aback by this comment. The title of the paper is "A cell-free platform for the prenylation of natural products and application to the efficient production of cannabinoids." In our view that is exactly what we report so it is hard to see how that is misleading. We were able to show that a cell-free system of 25 enzymes could be used to prenylate aromatic polyketides and used it to make cannabinoids. Indeed the work required considerable engineering well beyond engineering NphB mentioned by the reviewer. First, we had to re-engineer the pathway to accommodate aromatic compounds that were inhibiting the PDH enzyme. We then optimized the system parameters (we did not emphasize optimization since it is rather dull and specific to the particular system, but still considerable work). We then completely re-engineered NphB, completely altering its specificity and improving its catalytic efficiency by 1000-fold. This is not trivial and the entire point was to make the entire system work, not "just" engineer an enzyme. We then discovered that the system was being inhibited by CBGA accumulation and developed a flow system for removal of the product. The fact that we were able to identify the weak links in the system and re-engineer them so rapidly is a major benefit of the cell-free approach. We would like to emphasize that it is not at all obvious that one can build such complex systems involving 25 enzymes and many metabolites and make them work to generate the kinds of titers we report.

Perhaps we failed to sufficiently emphasize the considerable improvements that were made during the course of this work. We have attempted to rectify this by adding a few sections to the text.

We added some description of the optimization process to emphasize that things didn't just work right away:

> "Once we confirmed the PDH bypass improved 1,6 DHN titers, we began to optimize the new system as a general prenylation system. We varied co-factor concentrations, protein levels, and environmental conditions such as temperature and pH to identify the ideal set of conditions. Throughout this process we found that ATP, $NADP^+$, phosphate and NphB concentrations had the greatest impact on the final titer."

In the conclusion we added a summary of the progress we made overall:

> "When we started this project we were only able to produce 9 mg/L of CBGA using the monoterpene pathway developed by Korman et al. By introducing the PDH bypass and optimizing for co-factors, enzymes and environmental factors we were able to increase those titers to 132 mg/L. To improve titers further we engineered the NphB prenyltransferase, which further increased titers to 600 mg/L of CBGA. The final bottleneck was enzyme stability in the presence of CBGA, so by limiting the CBGA in the reaction vessel, we increased the titer to 1.25 g/L of CBGA, nearly a 140 fold improvement."

My main concenr is about the not sufficient analytics. Basic questions is not solved: Is the detected CBGA and CBVA correlated with the proposed structure? Zirpel et al, (2018) show

also various isomers and O-prenylation. It is very likely that the presented isoforms are O-prenylated products. Even by C-prenylation two mono prenylation sites are there. By MS we can exclude di-prenylation, but even that is not fully excluded. These questions can only be fully answered by NMR. Due to the high titers claimed, I suggest isolation and H/C NMR based structure elucidation before publication.

We agree with the reviewer regarding the NMR of CBGVA. Perhaps the reviewer missed the paragraph in the supplement on NMR characterization of the CBGVA product. We state that we compared the chemical shifts to previously published spectra. We have now also added the proton NMR spectra to the supplemental figures and also show it to the right.

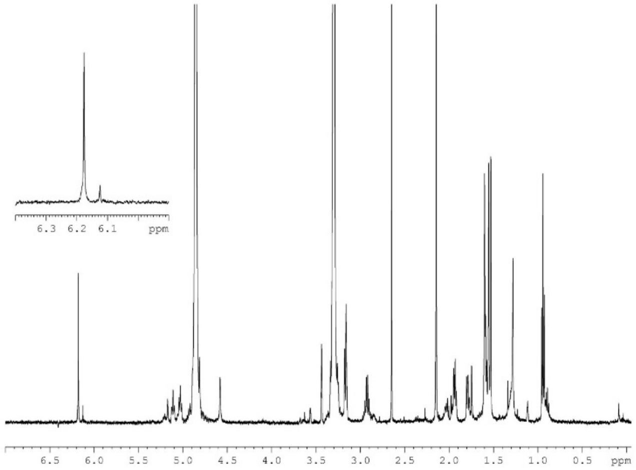

The identification of the CBGA product was greatly facilitated because we could obtain an authentic standard. First, our product has the same GC retention as the authentic standard. Second the MS spectrum is identical to the authentic standard. Third, the product can be converted into CBDA using a specific enzyme CBD synthase. Our data is also in agreement with Zirpel et al, where only two isomers were observed. Although Zirpel et al postulate four possible isomers, they report that they only observed two.

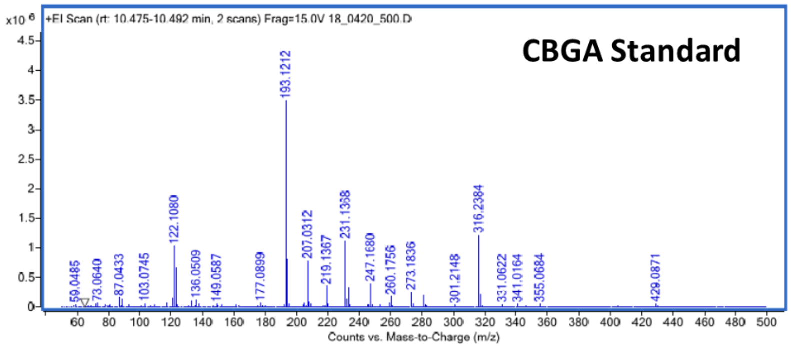

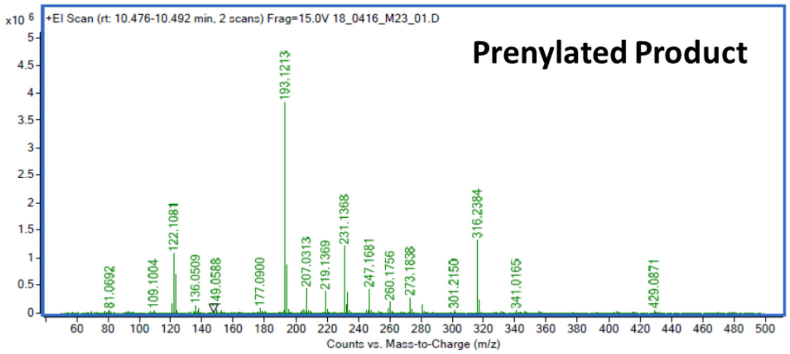

The mass spectra of the authentic CBGA standard and our product are shown to the right for comparison:

The proposed cell-free system is not innovative and has been used several times in ordinary labs determining enzyme kinetics. I am surprised to read here about an innovative system for industry, what is not true. The experimental design is a simple biotransformation that is by the way not fully characterized by bioengineers standards. Here a full process design is needed. Without that, I cannot see what is outstanding to be published in Nature Com.

We are mystified by this comment. The full complexity of the system we developed is perhaps best appreciated in Figure S1, but also can be seen in Fig. 1. Our cell free system employs 25 enzymes and associated cofactors and myriad metabolites! This is NOT a simple biotransformation. It would not be “ordinary” to use such a system for studying enzyme kinetics. Perhaps the reviewer is simply referring to the characterization of the NphB mutants which does utilizes standard assay methods, but this is only a part of the overall engineering of the cell-free system we describe in the paper.

The effect of a 1000 fold increased the activity of NphB is impressive. Mentioned facts like non-membrane binding and cytosolic activity are known. In the end, it is a protein engineering strategy in a rational way that is also not outstanding and a simple straightforward approach.

We respectfully disagree with Reviewer 1 regarding the engineered NphB. While the method may not be entirely novel, the properties of the mutant enzyme are. We did not simply increase binding generally of a non-native substrate, olivetolic acid. We improved specific binding in such a way that ONLY the correct position is prenylated while also increasing the activity 1000-fold. One might expect such changes in ligand binding to lead to inhibition of the enzyme. This is not the case. We have developed an enzyme that is a far more effective CBGA synthase than WT NphB, and it is a soluble enzyme which makes it far more suitable for the cell-free system than the native cannabis prenyltransferase, which is membrane bound. It was an essential step to achieving g/L scale quantities of the cannabinoids. We believe the many labs and companies working on cannabinoid production in cells will be excited to have this new enzyme. *The enzyme itself is an outstanding innovation in the cannabinoid production field in our opinion.*

Finally, as mentioned in the response to a previous comment, this paper is not solely about NphB engineering but in development and implementation of the whole system. The reviewer’s complete focus on NphB optimization does not come close to reflecting what we accomplished.

To sum up: Data provided are of high interest but are not outstanding to be published in Nature Communication. Cell-free production is no real option in the industry due to the fact that feeding all relevant precursors (GPP, OA etc) is too costly. There is no superior information about the question of energy supply and smart engineered substrate supply. Is it not clear where the focus is, is it about NphB optimization or cell free production.

We are again mystified by this comment. The entire point of building a system with 25 enzymes is to supply the expensive substrate GPP from inexpensive glucose! How is that not smart engineered substrate supply that the reviewer refers to? Moreover, the only way the entire system works is by using smart engineered supply of energy in the form of high energy cofactor regulation (see the purge valve system in Fig. 1).

The idea that you can’t build such a system on an industrial scale is an opinion that we intend to prove false and we have launched a company to do just that. What we do know is that the more well-worn cell-based approach has produced nothing like what we have been able to achieve in this work. We don’t claim that we have built an industrially viable system yet. Only that the approach has promise.

To hopefully emphasize these points we included more information about prior work in the cell-free area in the introduction:

“The synthetic biochemistry approach frees us from worrying about the toxicity of products and intermediates, affords rapid design-build-test cycles, precise control of all system components, and complete flexibility in pathway design. Nevertheless, building highly complex systems involving dozens of enzymes, associated cofactors and myriad metabolites on a large scale outside the context of the cell is an enormous challenge. One of the keys to making commercially viable cell-free systems is reducing enzyme costs by employing stable enzymes that can last for long periods of time. Recently Zhang and co-workers converted maltodextrin into inositol at a 20,000 L scale in a 5 enzyme system using thermophilic enzymes purified by simple heating step[1], demonstrating that at least simple cell-free systems can reach industrial scale. Another key requirement is designing systems that effectively generate and recycle high energy cofactors (ATP, NAD(P)H) so that they can be used many times. We have previously reported flexible purge valve and rheostats for the regulating the supply of reducing equivalents and ATP[2–4], allowing us to build systems that run for many days and produce high titers of isobutanol and terpenes.”

Reviewer #2 (Remarks to the Author):

In this manuscript the authors present a cell-free system for the production of the prenylated cannabinoid precursors, CBGA and CBGVA. They show that other molecules can be prenylated with the cell-free system, but to a lesser extent. In addition, the authors use computational modeling of protein-substrate interactions to identify enzyme mutations to improve kinetics in the cell-free reactions. The combination of protein design and synthetic biochemistry is timely, and the science addresses an important limitation in the synthesis of prenylated molecules. However, there are several areas where the manuscript falls short, which lessened my enthusiasm. One issue, for example, is that the title and abstract made me expect/want that the authors had made cannabinoids, but they did not (they made cannabinoid acids which would need to be decarboxylated to make cannabinoids). This paper was a step along the path to those molecules, but is really about prenylation. While I do not believe the manuscript in its current form is ready, if the authors address the concerns below, this manuscript could rise to the level needed to be accepted in a journal like Nature Communications.

Major Concerns:

- The authors argue that they have a cell-free system that makes cannabinoids, yet cannabinoids are not exactly made in the study. The authors make one cannabinoid acid, CBDVA, which is given only one sentence in the main text and the unquantified data is placed in the supplement. This was disappointing and made it feel like the authors oversell the data. The manuscript could be improved by carefully editing the text to accurately reflect the data presented.
- While this would require additional experimentation, I believe that the authors should make "therapeutically relevant cannabinoids" or their acids (THCA, CBDA, etc.) to warrant publication in this journal.

At the reviewer’s request, we have added production of CBDA to the results (see Fig. 4C). We actually had this data, but did not include it because, as outlined below, we don’t find this result particularly notable.

The reviewer is mostly concerned that we did not focus on cannabinoids that he/she deems important. We would argue that what the reviewer might consider "interesting" cannabinoids may well be just historical bias. In particular, THC and CBD have been easy to obtain from plants in the past and therefore are the best studied. That does not mean other cannabinoids won't prove to have interesting therapeutic properties. For example, the precursor CBGA that we produce in high titers in this work is being tested for ameliorating glaucoma. As more cannabinoids become readily available using systems like we describe here, the full panoply of biological effects of diverse cannabinoids may be revealed.

We would also argue that what the reviewer calls "cannabinoid acids" ARE cannabinoids. "Cannabinoid acids" are in fact what plants make. The acids are either decarboxylated spontaneously over time or more rapidly by heating (smoking). That's why people smoke marijuana. The decarboxylated forms are in essence a fortuitous degradation product. There is in fact no *biochemical* pathway to making the decarboxylated forms. Moreover, obtaining the decarboxylated forms is a trivial heating step. In our opinion the title is completely correct and appropriate. As discussed below we have added text to explain this better.

We understand where the reviewer is coming from, but we believe that the reviewer simply has a different perspective on what is important. Our focus in this paper was on delivering the *hard* step, i.e., making the CBGA and CBGVA, the precursors to many common and rare cannabinoids. Once you have CBGA and CBGVA you can make many other cannabinoids in a single enzymatic step and *this has already been demonstrated effectively in prior work*. Adding CBD synthase or THC synthase to CBGA to make CBDA or THCA is simply not novel or interesting. It's a trivial enzymatic step and has been done and published. Why is it important to do it yet again? What we have done is much more interesting and important in our opinion.

It has not been shown that CBGVA can be converted to CBDVA using CBD synthase, so we added that novel property to the paper.

We have re-written the section on converting CBGA and CBGVA to other cannabinoids to hopefully make our view more clear:

"To illustrate the production of other cannabinoids from the central cannabinoids CBGA and CBGVA, we employed CBDA synthase to convert CBGA into CBDA and CBGVA into CBDVA. Conversion of CBGA into CBDA has been demonstrated by several groups[5–8]. In our case, we simply transferred the nonane overlay containing CBGA to an aqueous solution containing CBDA synthase, and indeed we were able to convert CBGA into CBDA at a constant rate of 14.4 ± 0.8 mg L^{-1} hr^{-1} mg total $protein^{-1}$ over the course of 4 days converting 25% of the CBGA added to CBDA (Fig. 4C). To our knowledge it is not known whether CBGVA can be converted into the rare cannabinoid CBDVA using the CBDA synthase. So, we added CBGVA, extracted from the cell-free system, to a reaction containing CBDA synthase. CBDVA was produced (Fig S4) by CBDA synthase at a rate of 7.1 ± 0.1 mg L^{-1} hr^{-1} mg total $protein^{-1}$ for 24 hours. We note that the cannabinoid acids can undergo spontaneous decarboxylation or heat induced decarboxylation to form additional bioactive cannabinoids cannabidiol (CBD) and cannabidivarin (CBDV). Thus, our system provides opportunities for ultimately producing a wide-variety of cannabinoids."

- Line 91, the authors state that Enzyme assays revealed that PDH is inhibited by 1,6 DHN and other molecules. What about the panel of molecules used in Fig 2C? The authors should show PDH activity for all these molecules. In addition, Fig 2A seems to suggest that PDH activity might be improved by the addition of OA, their molecule of interest. This would suggest that they

shouldn't switch to the bypass system. Can the authors please clarify and did they try the final syntheses without the bypass? What are the results?

We thank the reviewer for this comment as we now realize that we should have included additional data on OA inhibition. We originally showed data for which all the substrates were at 1 mM. At these concentrations OA does not inhibit PDH, but when we go to 5 mM, we do see significant inhibition.

Our goal was to develop a general prenylating system and we discovered early that PDH inhibition was going to be a problem for at least some aromatic substrates. Thus, we employed the PDH bypass. But there is another advantage of the bypass system that we failed to emphasize in the original version of the paper: it avoids the huge PDH complex, which is difficult to isolate. Thus, once we knew that the bypass worked we stuck with it.

At the reviewer's request, we went back to look at inhibition of the additional compounds listed in Fig. 2C, but this proved technically challenging. Either the background absorbance at 340nm was too high, or the molecule was not soluble above 0.5 mM. While it might be possible to obtain the data in another way, we feel that additional efforts are not warranted since we don't want to go back to the PDH system for several reasons.

- Is the PDH bypass different than the author's previous work? Why wouldn't the authors start with the bypass system rather than showing us the PDH is less efficient? This doesn't seem like a new finding and seem supplemental to the stated goals of their manuscript.

The PDH bypass is different from our previous work and is therefore NOT supplemental to the stated goals of the work. Moreover, it provides an alternative approach that could be used in other cell-free systems if desired.

We have re-written the description of the development and optimization of the bypass system to hopefully clarify the points raised by the reviewer:

> "Enzyme assays revealed that pyruvate dehydrogenase (PDH) was inhibited by 1,6 DHN, as well as olivetol, resveratrol, and olivetolate (Fig. 2B). Therefore, to engineer a general prenylation system, we sought to eliminate PDH.
>
> To remove the need for PDH, we implemented a novel PDH bypass (Fig. 1). In the new PDH bypass, pyruvate is converted to acetyl-CoA using a pyruvate oxidase (PyOx) to produce acetyl-phosphate followed by the action of acetyl-phosphate transferase (PTA). The PDH bypass had two advantages. First, PDH is a large enzyme complex that is difficult to work with, so bypassing PDH streamlines enzyme production. More importantly, initial experiments revealed that the bypass is not subject to the inhibition seen at higher concentrations of 1,6 DHN. Once we confirmed the PDH bypass improved 1,6 DHN titers, we began to optimize the new system as a general prenylation system. We varied co-factor concentrations, protein levels, and environmental conditions such as temperature and pH to identify the ideal set of conditions. Throughout this process we found that ATP, $NADP^+$, phosphate and NphB concentrations had the greatest impact on the final titer. As shown in Fig. 2A, when we employed the PDH bypass, we found a 4-fold increase in titers of 5-prenyl-1,6 DHN when starting with 5 mM 1,6 DHN (Fig. 2A). When utilizing the PDH bypass system, approximately 50% of 1,6 DHN was converted in 24 hours, reaching a final titer of 705 ± 12 mg/L (Fig 2B)."

- Figure 2 is not properly put together. The legend doesn't correspond with the figures shown. For instance , I believe that 2A is described as 2B in the legend and vice versa. In addition, throughout the text these figures and their panels are not properly referenced making it difficult to read the manuscript. For instance, line 92 should be 2A not 2B; line 98 should be 2D not 2C. Careful editing needs to be carried out.

We thank the reviewer for pointing this out and apologize for the mix-up, which occurred when we were working to shorten the text. We have corrected the figure arrangement in order to reflect the figure captions and the manuscript text.

- Figure 2C tests aromatic substrates with 3 different prenyl transferases in the bypass system. The authors should test these substrates and transferases under the PDH system as panel 2A suggests that for some of these aromatics it might work better.

As noted above, there is actually no reason to think the PDH system would be better for any of the substrates. Nevertheless, we acknowledge that it might be possible, but that would be part of an optimization process for a particular substrate. In this work we present two alternatives that could be used if one were working on a particular substrate. No doubt for any other substrate, considerable optimization would be required as indicated by the extensive work we put into optimizing cannabinoid production. In general the bypass will be easier to implement than the PDH system, but optimization will be specific to each system.

In addition, the asterisks denote not determined in the figure. Just to be clear this means the chromatography had no distinguishable peak or these weren't tested?

The asterisks denote that the concentration prenyl-product was too low to quantify. We updated the figure legend accordingly with the following: (* indicates titer too low to be measured)

- Please be clearer in the legend that Figure 2D shows two different reaction setups in that different substrates are added. Presenting this in one graph makes it look like in one reaction you made prenylated DHN and CBGA.

The legend now reads: “Production of 5-prenyl-1,6 DHN (blue trace) over time compared to a <u>separate</u> reaction to produce CBGA (green trace).”

- Should ROSETTA be in all caps?

We are not sure if there are defined rules, but it is common to capitalize program names. In any case, we have changed it to Rosetta. Thank you.

- Line 112, a side-product is mentioned but the specifics should be stated. Did you observe side-products in the data? Can the data be provided?

Thank you for pointing this out. Production of the side product of the wild-type NphB enzyme is shown in Fig. 3C and identified previously as the 2-O-GOA isomer. We now explicitly state this in the text:

“As shown in Fig. 3C, WT NphB produces CBGA, but the dominant product is a prenylated side-product, 2-O-geranyl olivetolate, whereas M25 makes CBGA almost exclusively.”

- Lines 117-126 describes several conclusions drawn from data that is not referenced. I believe I was supposed to look at Figure S2. This should be a main body figure as the engineering of this enzyme is one of the more significant findings from this study.

We apologize for not making the appropriate references to supplemental figures in the text. This has now been corrected.

- Line 132, the natural prenyltransferase is mentioned. What is its activity?

We added the only citation we could find regarding the activity. The activity per mg of the prenyltransferase enzyme is unknown, however the Km for GPP is reported to be 2000 uM where the NphB Km for GPP is reported to be 130 μM.

We adjusted the text to read: "Overall, the designed enzyme is a much more effective CBGA synthase than WT NphB, and is easier to work with than the natural cannabis prenyltransferase, which is a membrane bound enzyme.[9,10]"

- Figure 4A should show WT NphB - CBGVA as well in order to compare. Also I believe this should be referenced on line 138.

We fully characterize and compare kinetic parameters for the WT and mutant NphBs in Table S5. Clearly, WT NphB is a horribly slow enzyme for both olivetolic and divirinic acid (< 1 turnover every three hours!). That's why we went to all that trouble to fix it. It is unclear to us what more would be learned by using a terrible enzyme in the full system when we have a good alternative. We added the reference as requested.

- Figure 4B is a schematic and the measured data should be shown alongside it. It is mentioned that 1.25 g/L of CBGA was made, but a time course similar to 4A should be shown. In addition, the whole point of this more complicated reaction setup was to alleviate precipitation of enzymes. Are there no longer preciptates in the reactions? How long do the reactions now last? This data should be provided.

We agree that a time course would be nice, but alas, this is a much more difficult experiment than it might appear. The substrate is found in three different layers (the reaction, the overlay and the trap). Thus, measuring the final product requires dismantling the entire set up and assaying each layer. It is therefore a single point assay system and we can only set up one reaction at a time. The point of developing the system was simply to show that we could increase titers by removing product as it is made and that is clearly shown by the results. A time course would not change that primary point.

We added a comment about the precipitation of enzymes in this experimental set up in the main text:

> "The flow system indeed improved the final titers to 1.25 ± 0.07 g/L, however enzyme precipitation still occurred at about 24 hours."

- Several of the supplementary figures (i.e. Figures S5-S10) are not, but should be, referenced in the text.

We apologize for the error and the citations have been added.

- The authors should cite their previous work on line 74.

We agree. Done.

- The authors should include exact conditions of their reactions or point to the methods section. Line 88 just says excess glucose.

Thank you. We adjusted the text regarding reaction conditions. "Up to ~400 mg/L (1.3 mM) of prenylated product was obtained from 2.5 mM 1,6 DHN."

Reviewer #3 (Remarks to the Author):

This article aims to construct cell-free enzymatic prenylating system for better production of important medicinal compounds. The cell-free system could break through the obstacles caused by membrane barriers, substance toxicities, etc. The approach studied in this article could provide a robust platform for producing chemicals that are hard to be made in living cells. This study is very interesting and also useful, but as currently presented, this manuscript needs to be further improved.

1. Glycolysis module, Acetyl-CoA module, and mevalonate module have been studied by many other researchers. Are there any innovative or obviously different research contents in this study?

Certainly many of the individual enzymes and pathways are known, but the trick is figuring out how to put them together so they work well in a cell-free system, appropriately generating and recycling high energy cofactors to continuously produce high titers of product.

2. Are there any other possible methods for PDH bypass, besides two enzymes PyOx and PTA?

Yes. For example, we have previously described one way as part of the PBG cycle (Opgenorth, et. al. *Nature Chem. Biol.* **12**, 393–395 (2016)). But the PBG pathway doesn't generate ATP so it could not be used to drive the mevalonate pathway. In a separate work, Krutsakorn, et. al,(*Microbial Cell Factories* **12**, 91 (2013)) decarboxylate pyruvate to form acetalydehyde which is then converted to acetyl-CoA through the action of a Co-A acylating acetaldehyde dehydrogenase, but again it would not generate ATP. There certainly may be other ways to do it that we hope will be described in the future.

3. The highlights of this article look like the construction of PDH bypass and the engineering of NphB. The depth of this cell-free platform needs to be increased, such as addressing the key challenges in this research field. More insightful information needs to be presented.

Thank you for this comment. While many of the challenges and promise of cell-free work have been reviewed and addressed in prior work, and we can't present a review here, we should have included more of the background in the introduction. Most people would consider simply putting together such an enormously complex system of 25 naked enzymes and different

cofactors that could operate for long periods of time, producing the kinds of titers we report here, an intimidating challenge. We have now re-written the introduction to emphasize these points and refer to prior work as follows:

"Here we propose an alternative biological approach to prenylated natural product biosynthesis using a cell-free enzymatic platform we call synthetic biochemistry, which has shown great promise for the production of bio-based molecules[12–18]. The synthetic biochemistry approach frees us from worrying about the toxicity of products and intermediates, affords rapid design-build-test cycles, precise control of all system components, and complete flexibility in pathway design. Nevertheless, building highly complex systems involving dozens of enzymes, associated cofactors and myriad metabolites on a large scale outside the context of the cell is an enormous challenge. One of the keys to making commercially viable cell-free systems is reducing enzyme costs by employing stable enzymes that can last for long periods of time. Recently Zhang and co-workers converted maltodextrin into inositol at a 20,000 L scale in a 5 enzyme system using thermophilic enzymes purified by simple heating step[1], demonstrating that at least simple cell-free systems can reach industrial scale. Another key requirement is designing systems that effectively generate and recycle high energy cofactors (ATP, NAD(P)H) so that they can be used many times. We have previously reported flexible purge valve and rheostats for the regulating the supply of reducing equivalents and ATP[2–4], allowing us to build systems that run for many days and produce high titers of isobutanol and terpenes."

4. How about the solubility of all expressed enzymes in this study?

All the enzymes could be concentrated to >10 mg/ml.

5. Have authors tried basic reaction parameters such as different temperatures and different pH values?

Thank you for this comment. Yes we vary many parameters during optimization, but it is a rather rote process and not generally interesting because it is highly specific to the particular system. Nevertheless, we should at least note that things don't just work fantastically right away. We have added the following text to simply indicate this:

"Once we confirmed the PDH bypass improved 1,6 DHN titers, we began to optimize the new system as a general prenylation system. We varied co-factor concentrations, protein levels, and environmental conditions such as temperature and pH to identify the ideal set of conditions. Throughout this process we found that ATP, $NADP^+$, phosphate and NphB concentrations had the greatest impact on the final titer. "

6. Cofactors and energy substrates have significant impacts on pathway activities. Have authors done any theoretical calculation or experiments for better control of cofactor and energy levels?

Yes indeed. High energy cofactor maintenance and recycling are critical for making cell free systems work. These issues were attacked in prior work in which we developed regulatory systems such as the purge valve used here. These are now better highlighted in the introduction (see above). The prior innovations are part of the reason the current system works.

7. Line 156: Two periods.

Fixed. Thank you.

8. Results in this study should be compared with previous studies by other researchers. Please indicate how the results relate to expectations and to earlier research.

This is detailed in the introduction. However, we re-worked the introduction to make it more clear how this research differs from what's previously been done.

"Microbial production is a useful alternative to natural sourcing for prenyl natural products, but it comes with many challenges. Prenyl natural products like prenyl-naringenin, prenyl-resveratrol and cannabidiol (CBD) are derived from a combination of fatty acid, polyketide and terpene metabolic pathways. Therefore, high-level biosynthesis requires re-routing long, essential and highly regulated pathways. Many groups have engineered microbes to produce the polyketide intermediates, like naringenin, resveratrol and olivetolate, but the titers are limited (110 mg/L, 391 mg/L and 80 mg/L respectively).[19,20] The titers for the prenyl-products are expected to be even lower because GPP is toxic to cells, creating a significant barrier for high level microbial production.[21] So, in spite of intense interest, to our knowledge there are still no published reports of the complete biosynthesis of prenyl-flavonoids, prenyl-stilbenoids or cannabinoids in recombinant microbes.

The most effort has been directed to the cannabinoid field. Two groups have produced the polyketide intermediate, olivetolic acid (OA) at low levels in yeast (0.5 mg L^{-1}) or *E. coli* (80 mg L^{-1}), but did not prenylate OA or produce a cannabinoid from the biosynthesized OA[20,22]. In other work, THC was produced from either exogenously added geranyl-pyrophosphate (GPP) and OA in a two enzyme pathway[9] or from cannabigerolic acid (CBGA) using a single enzyme [15]. However, it is unclear how GPP or CBGA could be obtained at sufficient levels for economic production due to the high cost of these molecules."

9. More application examples need to be provided to prove the effectiveness of this cell-free prenylation platform.

We included 8 examples to show that the system works for a variety of substrates. We describe full optimization for one of them (cannabinoids) and it was a ton of work. This illustrates that meaningful titers can be obtained, but doing full optimization on more examples is simply too much for one paper.

REVIEWERS' COMMENTS:

Reviewer #1 (Remarks to the Author):

The authors have well answered to my comments and most of the problems and criticism have been sorted out. I still believe that the topic of cell-free biotransformation is not novel and does reach the level of novelty to be published in Nature COm, but this is an Editor decision.

Reviewer #2 (Remarks to the Author):

I thank the authors for their revised manuscript, which mostly addresses the questions I had. I do very much like the concept of this paper, but was surprised by the authors' tone in their response to reviewers.

Reviewer #3 (Remarks to the Author):

This manuscript has been well revised. This work would interest many reseachers in this research field. So I suggest the acceptance.

Response to Reviewer comments

Reviewer #1 (Remarks to the Author):

The authors have well answered to my comments and most of the problems and criticism have been sorted out. I still believe that the topic of cell-free biotransformation is not novel and does reach the level of novelty to be published in Nature COm, but this is an Editor decision.

Reviewer #2 (Remarks to the Author):

I thank the authors for their revised manuscript, which mostly addresses the questions I had. I do very much like the concept of this paper, but was surprised by the authors' tone in their response to reviewers.

Reviewer #3 (Remarks to the Author):

This manuscript has been well revised. This work would interest many reseachers in this research field. So I suggest the acceptance.

RESPONSE: We thank the reviewers for their help in improving the manuscript.
